# In-Depth Multi-Approach Analysis of WGS Metagenomics Data Reveals Signatures Potentially Explaining Features in Periodontitis Stage Severity

**DOI:** 10.3390/dj13120590

**Published:** 2025-12-08

**Authors:** Ignat V. Sonets, Iulia S. Galeeva, Danil V. Krivonos, Alexander V. Pavlenko, Andrey V. Vvedenskiy, Anna A. Ahmetzyanova, Karen A. Mikaelyan, Elena N. Ilina, Oleg O. Yanushevich, Zalina E. Revazova, Elena I. Vibornaya, Galina S. Runova, Vasiliy V. Aliamovskii, Irina S. Bobr, Madina O. Tsargasova, Ekaterina I. Kalinnikova, Vadim M. Govorun

**Affiliations:** 1 Department of Mathematical Biology and Bioinformatics, Research Institute for Systems Biology and Medicine, Moscow 117246, Russia; 2 Moscow Center for Advanced Studies 20, Kulakova Str., Moscow 117246, Russia; 3 Department of Clinical Dentistry, FSBEI HE “ROSUNIMED” of MOH of Russia, Moscow 127473, Russia

**Keywords:** dentistry, periodontitis, metagenome, whole genome sequencing, metabolic pathways, analysis of variance

## Abstract

**Background**: Periodontitis is a chronic inflammatory disease mostly associated with *Porphyromonas gingivalis* infection and characterized by progressive destruction of the supporting structures of the tooth, including the gingiva, periodontal ligament and alveolar bone. However, the impact of other members of the periodontal microbiome on stage of the severity of the periodontitis remains largely uncharacterized. **Methods**: This exploratory study employs whole-genome shotgun (WGS) metagenomics to characterize the periodontal microbiome in patients suffering from mild and severe periodontitis, aiming to identify microbial signatures linked to disease severity via analysis of taxonomic composition, predicted metabolic pathways and metagenome-assembled genomes (MAGs). After initial selection, 28 adult patients with a computer tomography (CT)-confirmed diagnosis of mild and severe stage of periodontitis from 2 clinics were included in the research project. **Results:** Taxonomic analysis confirms the presence of various commensal and pathogenic bacteria detectable at the species level, especially belonging to so-called “red, orange and green periodontal complexes”—*P. gingivalis*, *T. forsythia*, *C. rectus*, and *Capnocytophaga* spp. that may contribute to disease heterogeneity. The conducted investigation suggests that non-microbial factors such as cardiovascular diseases and antibiotic usage in the last 6 months prior to the hospital admission could explain variance of disease progression and impact on severity. Analysis of microbial functional composition revealed metabolic traits showing positive correlations with severe stage of periodontitis. Robust network analysis suggested interactions between pathogenic bacteria of the red complex and other members of the periodontal microbiome. **Conclusions:** These findings underscore the multifactorial nature of periodontitis pathogenesis, highlighting the need for integrated approaches combining microbial, host, and environmental data to unravel drivers of disease progression. The study provides a foundation for future large-scale investigations into personalized diagnostic or therapeutic strategies.

## 1. Introduction

Periodontitis is a chronic inflammatory disorder that causes progressive destruction of tooth-supporting structures, including the gums, periodontal ligament, and alveolar bone. Clinically, it progresses from mild stages (gingival inflammation, pocket formation) to severe forms involving irreversible bone loss, tooth mobility, and as final result, tooth loss [1,2,3]. Globally, severe periodontitis affects about 11% of adults, making it the sixth most prevalent human disease [4]. Beyond oral health, periodontitis is linked to systemic conditions such as diabetes mellitus [5] and could play a role as a risk factor for cardiovascular diseases [6]. These associations underscore its role as a modifier of systemic inflammation, driven by dysbiotic microbial communities that translocate into circulation via ulcerated periodontal pockets [7].

The oral microbiome comprises over 1000 bacterial species and it is the second most diverse microbiome in humans [8], and it is composed of bacteria alongside archaea, fungi, and viruses, forming complex biofilms that interact dynamically with the host immune system [9]. Environmental stressors (e.g., poor oral hygiene, smoking, and other factors) trigger dysbiosis, favoring pathobionts such as *Porphyromonas gingivalis*, *Tannerella forsythia*, and *Treponema denticola—*collectively termed the “red complex” [10]. These taxa produce different virulence factors that degrade host tissues, activate destructive immune responses, and disrupt microbial homeostasis [7,11]. However, a significant knowledge gap exists regarding the functional impact of the broader microbial community in periodontal pockets. The specific role of these microbes—as drivers or suppressors of inflammation—is not definitively characterized, and the available literature presents conflicting evidence. This ambiguity poses a substantial obstacle to advancing both fundamental research and the creation of effective clinical applications.

In recent years, high-throughput sequencing has become one of the most used methods to investigate microbiome composition. The 16S rRNA metagenomic sequencing is a widely used technique in metagenomics studies and remains standard procedure for most research applications. While cost-effective for bacterial taxonomic profiling, it suffers from some inherent limitations that may hinder comprehensive microbiome analysis [12]. Whole-genome shotgun (WGS) metagenomics addresses these limitations by sequencing whole-length microbial DNA in a sample, bypassing PCR amplification biases, and providing comprehensive genomic insights [13,14].

In our study, we investigated the periodontal microbiome composition in 30 outpatients with periodontitis associated with *P. gingivalis* by WGS metagenomics. To assess the severity of the disease, we utilized computed tomography (CT) scans to evaluate the degree of tissue damage according to 2018 AAP/EPF Classification of Periodontal and Peri-Implant Diseases. These assessments enabled us to split the patients cohort into two severity groups (mild and severe stages of periodontitis) based on the severity of their disease course. Our objective was to identify microorganisms that are associated with the severity of the disease by taxonomic classification, analyze relationships between the periodontal microbiome composition of patients that suffered with periodontitis and covariates, conduct alpha- and beta-diversity estimations, to analyze metabolic pathways and link their impact on different stages of periodontitis, and to conduct differential abundance testing. Furthermore, we examined the variations in the structure of the microbial interaction network between these two groups. We anticipate that the findings from our study will enhance the understanding of the mechanisms underlying the impact of clinical manifestations of periodontitis on the human oral cavity microbiota.

## 2. Materials and Methods

### 2.1. Study Cohort

The study included 30 patients with laboratory-confirmed diagnosis of periodontitis and clinical manifestations of it, who were appointed and treated at the Clinical Center of Dentistry and at the “Center of Dentistry and Maxillofacial Surgery” Clinic of FSBEI HE “ROSUNIMED” of MOH of Russia in October of 2023. Samples were obtained from 15 patients with mild cases of periodontitis and 15 patients with severe cases of periodontitis upon their visit to the dentist. PCR testing of biomaterial for presence of *P. gingivalis* was conducted to confirm ongoing periodontitis infection. Patients were assigned into cohort groups (mild and severe periodontitis) based on results from CT scanning. According to the 2018 AAP/EPF Classification of Periodontal and Peri-implant Diseases, bone loss was assessed using cone-beam computed tomography (CBCT). Radiographic bone loss (RBL) relative to the roots of teeth with medium periodontitis (stage II grade B) was determined at the level of the coronal third of the root (RBL 15–33% horizontally). For severe periodontitis (stage III grade B and stage IV grade B), it was determined at the level of the middle third of the root or more (RBL ≥ 50% horizontally), with vertical bone loss ≥ 3 mm.

All the patients signed statements of informed consent to participate in the study. After excluding 2 samples due to insufficient sequencing results, the resulting group consists of 13 patients with mild periodontitis and 15 patients with severe periodontitis. There were 14 male patients and 14 female patients ranging from 36 to 73 yrs old (median for males 46 years and 57 years for females). The metadata were collected based on questionnaires given to the patients which included criteria such as prior treatment of periodontitis, antibiotics usage, patient’s age and gender, smoking status, and other factors. A sample sheet of the questionnaire is presented on Supplementary Table S1. Descriptive statistics of quantitative metadata variables are presented in Table 1.

**Figure 1:**
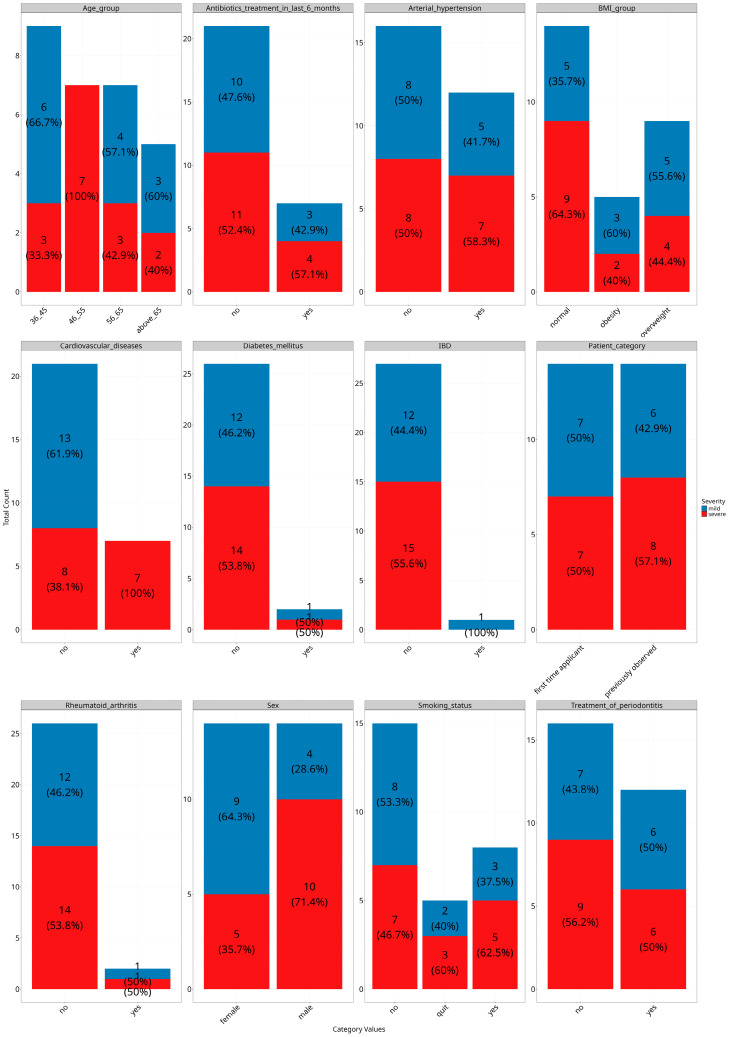
Study cohort overview of and distribution of the metadata by stage of severity of periodontitis group (*Y*-axis for all subplots are number of counts).

A detailed graphic overview about the study cohort is shown in Figure 1.

Patients fulfilled all the following study inclusion criteria:

1. Men and women aged 18 years and older with a confirmed diagnosis of “Chronic periodontitis (K05.3) newly diagnosed or previously treated in the acute stage” with confirmed *P. gingivalis* presence by PCR of oropharyngeal swab;
2. Mild and severe dynamics of the course of periodontitis according to CT scan;
3. Signed voluntary informed consent for participation in the study.

A patient was excluded from the study if the subject met at least one of the following exclusion criteria:

4. Antibiotics treatment in the last month prior to visiting a dentist;
5. Performing hygienic cleaning of teeth prior to visiting a dentist;
6. Absence of *P. gingivalis* DNA in the biomaterial according to the results of PCR testing.

All patients signed an informed consent form to participate in the study and the study was approved by the Inter-University Ethics Committee, protocol No. 03–23 of 16 March 2023.

### 2.2. Samples and Data Collection

The contents of 3 periodontal pockets of each of 30 patients were collected using a sterile curette. The collected biomaterial (periodontal pocket contents) was transferred to a single tube which was later processed as a single sample (i.e., pooled per sample) with 300 μL of TE buffer using a cotton swab (10 mM TRIS-HCl, 1 mM EDTA, pH = 8) and transported to the laboratory in a portable cooler with cool-packs within 1–5 h. DNA from the samples was either isolated upon receipt or frozen at −20 °C for subsequent isolation within 1–3 days.

### 2.3. WGS Sequencing

DNA isolation was performed using the Sorb-GMO-A kit (Syntol, Moscow, Russia). The concentration of the resulting DNA solution was determined using the QuDye HS kit (Lumiprobe, Moscow, Russia) on a Qubit™ 4.0 device (Thermo Fisher Scientific, Waltham, MA, USA). The concentration of the samples varied from 1.33 to 32 ng/μL. The presence of *P. gingivalis* DNA was confirmed by real-time PCR using the commercial “Gingipol” kit with fluorescently labeled probes (Lytech Co., Ltd., Moscow, Russia). Sample preparation was carried out using the Fast FS DNA Library Prep Set (MGI, Shenzhen, China) following guidelines from the kit manufacturer. Sequencing was performed on a DNBSEQ-G400 instrument (MGI, Shenzhen, China) using the DNBSEQ-G400RS High-throughput Sequencing Kit (FCL PE150 in paired-end mode 2 × 150 bp and dual barcode reading 2 × 10 bp. All 4 lanes of flowcell were used for sequencing and raw reads from lanes were later merged into one set of paired sequence files. All samples were sequenced in one run. Distilled water was used as a negative control during sequencing.

### 2.4. WGS Data Processing

Sequencing of given samples yielded on average 113 million reads per sample with a median of 106 million reads in the total reads number. However, a big part of these reads consisted of human data, around 60% on average. Based on Figure S1 and recommendations from [15] as well as results from [16,17], we decided to exclude 2 patients (MPAR_80 and MPAR_87) because the total number of non-human reads did not meet the criteria of 10 million reads minimum to be applicable for further analysis.

The following analysis was conducted using the same set of obtained FASTQ files. KneadData v0.12.0 (https://github.com/biobakery/kneaddata, accessed on 7 February 2025) was selected for QC filtering (MAPQ ≤ 15), auto removing adapters and primers, decontaminating and removing human reads using hg38 bowtie2 index file provided with KneadData. MetaPhlAn v4.0.6 [18] and its 29 August 2024 database was used for taxonomic profiling. HUMAnN v3.9 [19] and its ChocoPhlAn and UniRef90 databases were used for metabolic pathways prediction; non-bacterial pathways and incomplete and/or unidentified pathways were removed. MAGs analysis was performed using aforementioned tools:* de novo* assembling of sequencing data was performed via MEGAHIT v1.2.9 [20], de novo binning was conducted with MetaBAT2 v2.15 [21], reads were mapped with bwa v0.7.19 [22], MAGs quality assessment was made with CheckM v1.0.1 [23]. dRep v3.5.0 [24] was selected for MAGs dereplication, MAGs coverage estimation was conducted with InStrain v1.9.0 [25] and CoverM v0.7.0 [26] tools. MAGs were taxonomically annotated via gtdbtk v2.4.0 [27] and its r220 database [28].

The resulting dataset consists of 518 microbial species that belong to 94 families. Following quality control filtration of metagenome-assembled genomes (MAGs) based on criteria of ≥80% completeness and ≤10% contamination, 193 high-quality MAGs were retained. Metabolic pathways profiling with HUMAnN revealed 389 joint bacterial metabolic pathways across all samples.

### 2.5. Data Analysis

The statistical analysis of the microbiome data was conducted using R version v.4.4.3. Packages phyloseq v.1.5.4, speedyseq v.0.5.3, ggpubr v 0.6.0, microbiome v.1.3.2, vegan v.2.7-2, tidyverse v.2.0.0, DESeq2 v1.38.3 [29], NetCoMi v.1.2.0 [30] were used to perform subsequent analysis. Functions such as *core_members* and *prune_taxa* from microbiome and phyloseq packages were used to filter low abundant taxa from analysis with detection threshold = 1 and prevalence threshold = 0.1. The ggplot2 package (https://ggplot2.tidyverse.org, accessed 11 June 2025) was used for visualization.

Bacterial microbiome diversity was evaluated using alpha-diversity metric, specifically the Shannon index, calculated through the *plot_richness* function in the phyloseq R package. Beta-diversity was assessed using Bray–Curtis distance on compositional-transformed data (which was selected because most widely used and recognized dissimilarity metric, thus providing direct comparison to majority of results from other studies, as also providing ease of zeros handling), calculated with the *dist* function from the vegan package and visualized using the *plot_ordination* function from phyloseq. Statistical significance between groups was determined using the ANOSIM test. Results of beta-diversity analysis were visualized via principal coordinate analysis (PCoA). Permutational multivariate analysis of variance (PERMANOVA) was conducted to identify associations between microbial taxa and sample features using the *Adonis* function from the vegan package using Bray–Curtis distance on compositional-transformed data. To ensure that centroid differences were not caused by dispersion differences between groups, permutational dispersion analysis via *permdisp* function in vegan R package was conducted. VIF (variance inflation factor) correction was applied to address multicollinearity among covariates, which can lead to unreliable significance tests in PERMANOVA. Variables with VIF > 5 were removed to ensure that each predictor’s contribution to microbiome variation could be independently assessed without inflated type I error rates. Later models were adjusted by excluding variables with highest p-values. For differential abundance analysis, the DESeq2 package in R was used with the following formula ~’variable of interest’. For DESeq2, a significance cut-off BH-corrected *p*-value < 0.05, prevalence threshold = 0.1 and detection threshold = 0.01 and abs(Log2FoldChange) ≥ 1 cut-off were applied. The co-abundance networks of WGS species data were generated using the SPIEC-EASI algorithm with the Meinshausen–Bühlmann method implemented in functions from the NetCoMi package. The following parameters were utilized for generation: 25 lambda iterations, 100 bootstrap replicates, minimum lambda value of 0.001, and minimal stability edge frequency threshold = 0.8. Clusters of co-abundant species in the final network were identified using the Louvain method. Eigenvector centrality and degree centrality were used for defining hubs/keystone taxa (which are nodes with a centrality value above the empirical 95% quantile). Prior to net construction, data was filtered by prevalence threshold = 0.7 and abundance threshold = 0.1, thus providing an adequate taxa-to-sample ratio. Analysis to estimate associations between microbial metabolic pathways features and metadata variables was conducted using the MaAsLin2 package [31] using CPLM, LM and NEGBIN statistical models, data normalization using the TSS method for CPLM and LM models and TMM method for NEGBIN model, and BH correction method. The significance thresholds were set at a *q*-value  <  0.05 and abs(coef) ≥ 1, and the prevalence threshold was set at 0.1.

## 3. Results

### 3.1. Taxonomy of Periodontal Microbiome of Periodontitis Patients Infected with P. gingivalis

**Figure 2:**
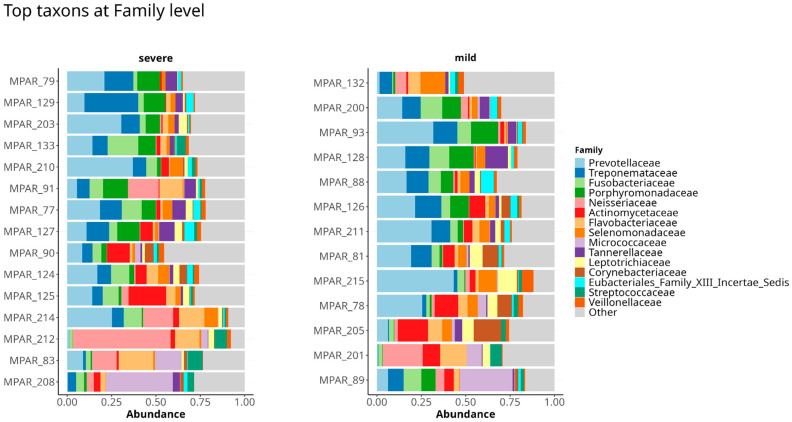
Top 15 abundant families present in sample data. Samples are grouped by stage of severity of periodontitis.

In total the 518 microbial species that belonged to 94 families were identified from 28 patients with varying degrees of periodontitis severity. The top ten predominant families were *Prevotellaceae*, *Treponemataceae*, *Fusobacteriaceae*, *Porphyromonadaceae*, *Neisseriaceae*, *Actinomycetaceae*, *Flavobacteriaceae*, *Selenomonadaceae*, *Micrococcaceae* and *Tannerellaceae* (Figure 2).

**Figure 3:**
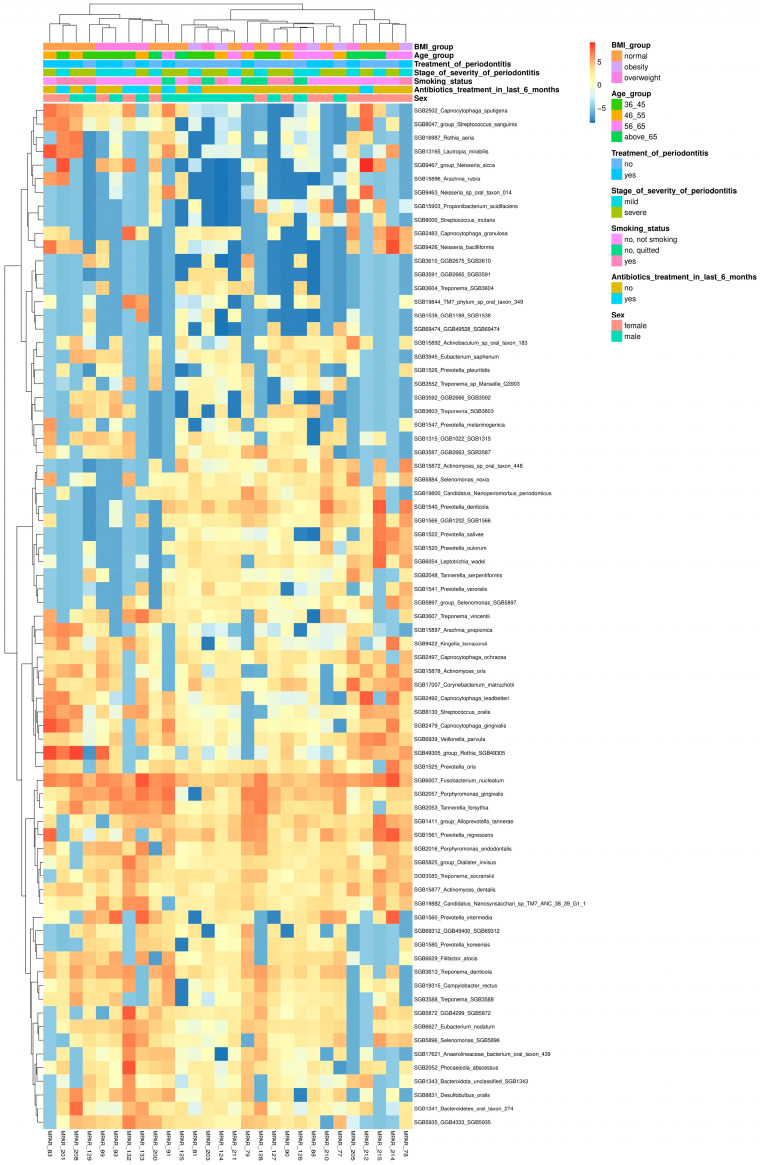
Heatmap showing the abundance of top 75 prevalent species in patients with mild and severe periodontitis. Read abundance is CLR normalized and Ward.D2 clustered by rows for better representation of patterns.

Comprehensive taxonomic profiles at genus and species levels are presented in Figures S2 and S3. Hierarchical clustering of the most prevalent species (prevalence threshold = 0.1) is shown in Figure 3.

Using WGS metagenomic data we managed to obtain the 193 high-quality MAGs, and their hierarchical clustering is shown in Figure S4. A total of 389 joint metabolic pathways across all samples were annotated with HUMAnN.

### 3.2. Alpha- and Beta-Diversity of WGS Metagenomics Data

**Figure 4:**
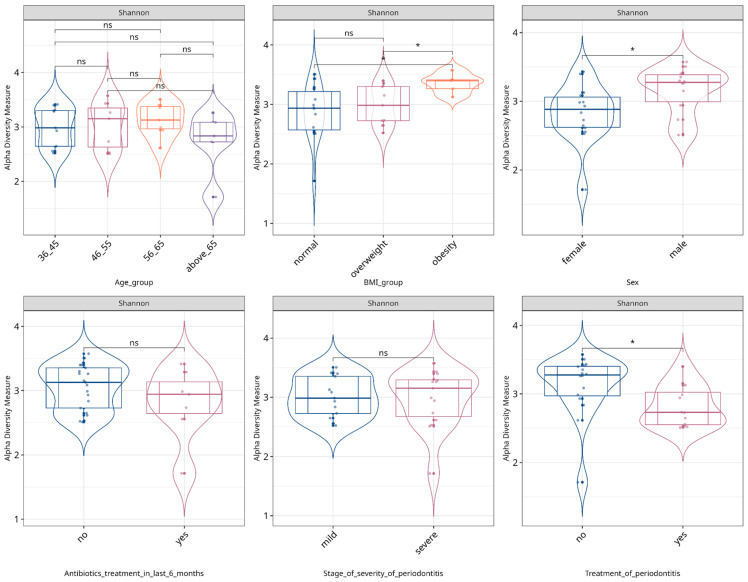
Alpha diversity by Shannon index. Box plots illustrate alpha diversity by Shannon index in bacterial microbiomes of 28 patient samples across different variables. Median values and interquartile ranges have been indicated in the plots. * indicates *p* < 0.05, representing levels of statistical significance"; ns means “not significant”.

Microbial alpha diversity, assessed using the Shannon metric, was utilized to examine changes in the periodontal microbiota community structure for patients categorized into mild and severe groups. We analyzed alpha-diversity across all covariates data collected via questionnaire. Similar analyses were conducted on both WGS data (Species level) and MAGs datasets. Conducted filtration (see Methods for functions core_members and prune_taxa) narrowed WGS dataset to 75 species and MAGs dataset to 143 MAGs. Figure 4 displays the outcomes, indicating that no statistically significant variations (Wilcoxon test) were observed in the alpha diversity indices between the two patients’ groups.

Figure S5 displays results of similar analysis implemented on MAGs dataset. Although we found no significant differences between patients with mild and severe periodontitis in their alpha-diversity we observed statistically significant differences between male and female patients—men tend to have more diverse periodontal microbiome composition. Across three BMI groups (normal weight, obesity, and overweight) patients with obesity had the most diverse periodontal microbiome. Notably, patients who had received periodontitis treatment showed reduced alpha-diversity compared to untreated individuals.

**Figure 5:**
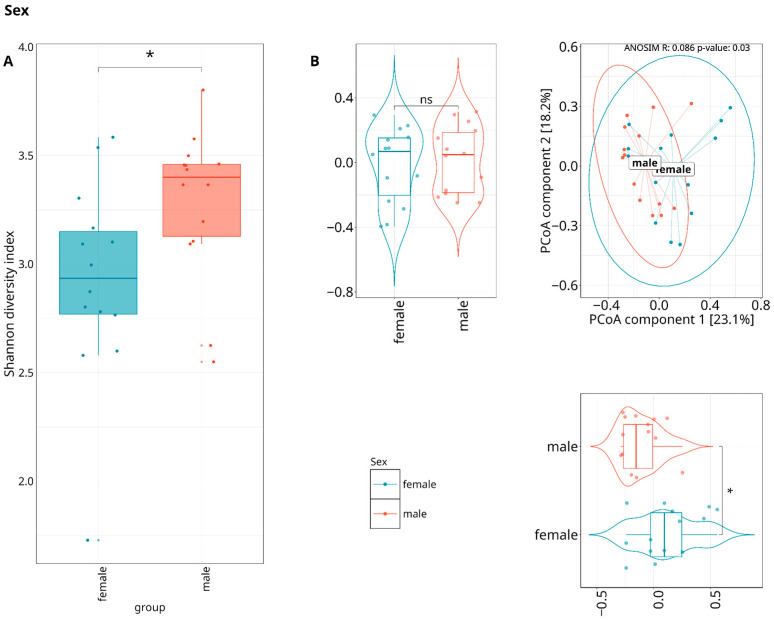
Composed Shannon index alpha-diversity (**A**) and PCoA plots (**B**) of beta-diversity estimated with Bray–Curtis distance and statistical test results (ANOSIM test) between MAGs composition of different sexes. Median values and interquartile ranges have been indicated in the plots. Violin plots are displaying statistical differences for each PCoA component. Ellipses are drawn around 95% confidence level based on a multivariate t-distribution. “*” indicates p < 0.05, representing levels of statistical significance; “ns” means “not significant”.

**Figure 6:**
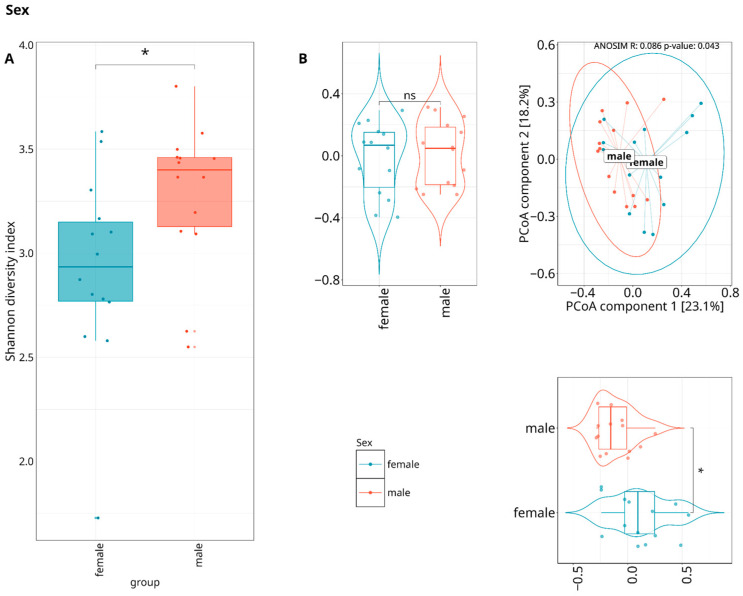
Composed Shannon index alpha-diversity (**A**) and PCoA plots (**B**) of beta-diversity estimated with Bray–Curtis distance and statistical test results (ANOSIM test) between metabolic pathways composition of patients with different sexes. Median values and interquartile ranges have been indicated in the plots. Violin plots are displaying statistical differences for each PCoA component. Ellipses are drawn around 95% confidence level based on a multivariate t-distribution. “*” indicates *p* < 0.05, representing levels of statistical significance; “ns” means “not significant”.

Beta-diversity was calculated by using the Bray–Curtis and Euclidean distances and visualized with a principal coordinate analysis (PCoA), showing no statistically significant variations (ANOSIM test) between the two severity groups across three datasets (species/MAGs/metabolic pathways); however, statistically significant differences were observed between sexes (Figure 5) on MAGs and metabolic pathways (Figure 6) level.

**Figure 7:**
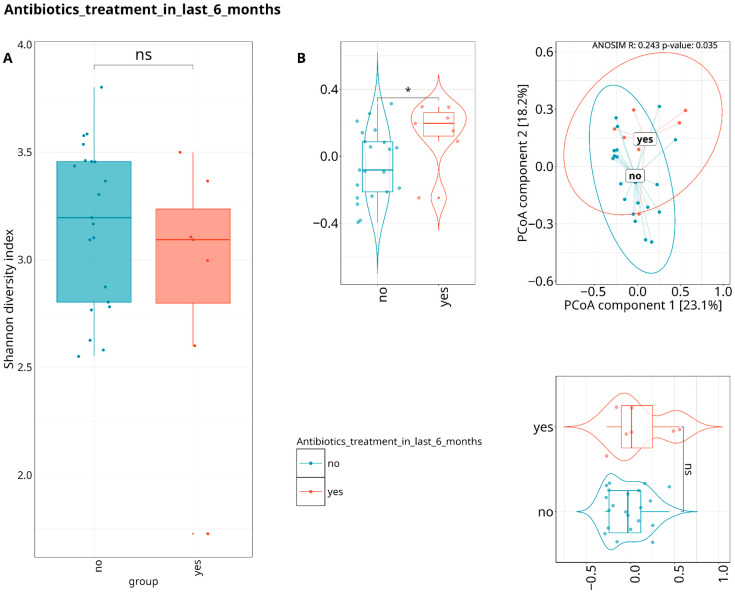
Composed Shannon index alpha-diversity (**A**) and PCoA plots (**B**) of beta-diversity estimated with Bray–Curtis distance and statistical test results (ANOSIM test) between WGS taxonomic composition of patients undergoing or not undergoing antibiotics treatment in the last 6 months before inclusion in the study cohort. Median values and interquartile ranges have been indicated in the plots. Violin plots are displaying statistical differences for each PCoA component. Ellipses are drawn around a 95% confidence level based on a multivariate t-distribution. “*” indicates *p* < 0.05, representing levels of statistical significance; “ns” means “not significant”.

On a species level, differences were detected between patients who underwent antibiotics treatment in the last six months prior to their visit to the dentist, showing profound effect on the periodontal microbiome composition after long periods of time (Figure 7).

Additional results showing alpha- and beta-diversity for covariates of interest are presented on Figures S6–S8.

### 3.3. Exploring the Relationship Between the Periodontal Microbiome and Covariates

**Figure 8:**
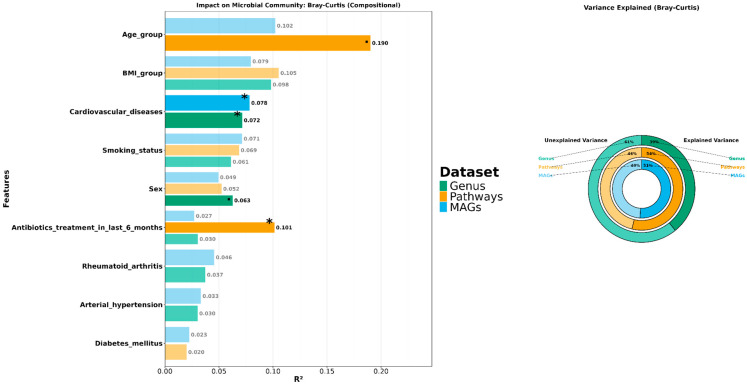
Metadata factors explain the variance in microbiome composition across 3 datasets (WGS data, MAGs, metabolic pathways). Asterisks indicate the level of significance with the following thresholds: (*) = 0.05, (.) = 0.1. R^2^ values for each covariate are reported on the plot. PERMANOVA was calculated using Bray–Curtis distance metric and compositional data transformation.

For testing the association between the microbiome and the clinically significant covariates of age group, stage of severity of periodontitis, IBD, diabetes, BMI group, hypertension, smoking status, rheumatoid arthritis, usage of antibiotics in last six months prior to sample collection, treatment of periodontitis, cardiovascular diseases, and arterial hypertension, we applied a PERMANOVA to all three of our datasets using both Bray–Curtis distance and compositional data transformation. (Figure 8).

VIF analysis excluded 2 clinically significant variables such as “treatment of periodontitis” and “patient category” due to high autocorrelation (VIF > 5) (Supplementary File S1). For the WGS dataset with taxa agglomerated by Genus level we found that covariates explain 39% of variance, among them the covariates “cardiovascular diseases” (R^2^ = 0.072) are statistically significant for explaining variance between patients. PERMANOVA analysis of predicted metabolic pathways (HUMANn pathways dataset) dataset showed that the covariate “antibiotics treatment in last 6 months” (R^2^ = 0.101) is a statistically significant driver of variance between samples; in total, all included covariates can explain 54% of variance. Speaking of MAGs dataset, we found that these covariates explained 51% of the periodontal microbiome taxonomic composition with the most extensive and statistically significant contribution coming from the “cardiovascular diseases” comparison. Additional testing made with permutational dispersion analysis for each of the datasets (PERMDISP) verified our findings, so the difference between centroids of variables’ groups that explain variance in PERMANOVA were not caused by difference in dispersion inside groups. (Supplementary Files S2–S4).

### 3.4. Analyzing Microbial Associations with Disease Severity in Relation to Variables and Comorbidities

**Figure 9:**
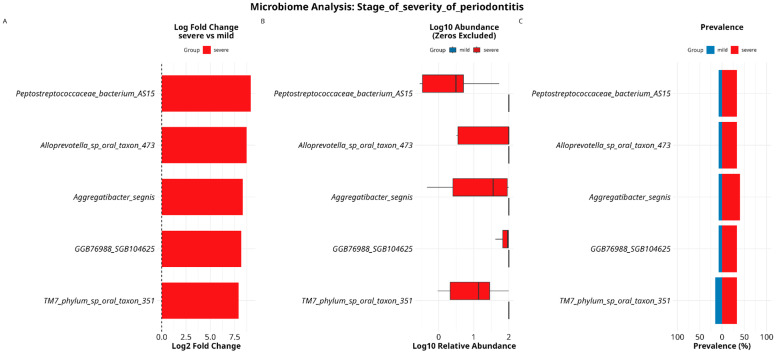
DESeq2 results (**A**) for differentially abundant taxa testing associations with stage of severity of periodontitis. Species associated with a milder course of disease are colored in blue; species associated with a more severe course of periodontitis are colored in red. Verification of results are made with calculating their log10 abundance (**B**) and estimating prevalence of these species (**C**).

We performed DESeq2 differential abundance analysis on species level, using “stage of severity of periodontitis” and “treatment of periodontitis” as a grouping factors, hence these covariates are some of the most important factors to estimate microbiome composition and its response to either disease stage progression or treatment effectiveness. As a result, 383 species and 143 MAGs were analyzed. Differential abundance analysis showed differences in the abundance of five species, all of them exhibited statistically significant associations with the severe degree group in the context of periodontitis: *Peptostreptococcaceae_bacterium_AS15* (*p*-adj = 0.014, logFC = 9.16), *Alloprevotella_sp_oral_taxon_473* (*p*-adj = 0.0204, logFC = 8.73), *Aggregatibacter segnis* (*p*-adj = 0.0204, logFC = 8.32), *GGB76988_SGB104625* (*p*-adj = 0.035, logFC = 8.18), and *TM7_phylum_sp_oral_taxon_351* (*p*-adj = 0.034, logFC = 7.88) (Figure 9).

**Figure 10:**
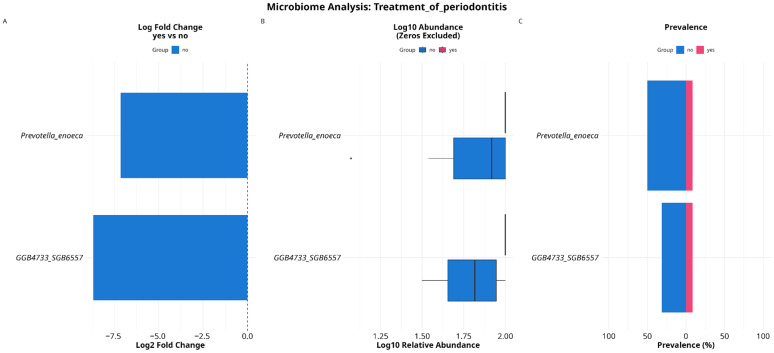
DESeq2 results (**A**) for differentially abundant taxa testing associations with antibiotics treatment. Species associated with prior treatment are colored in light-blue; species associated with absence of prior treatment are colored in pink. Verification of results were made by calculating their log10 abundance (**B**) and estimating the prevalence of these species (**C**).

When using the “treatment of periodontitis” as a grouping factor, we discovered differences in the abundance of two species, both of them were associated with absence of treatment administration: *Prevotella enoeca* (*p*-adj = 0.047, logFC = 7.14) and *GGB4733_SGB6557* (*p*-adj = 0.024, logFC = 8.69) (Figure 10).

**Figure 11:**
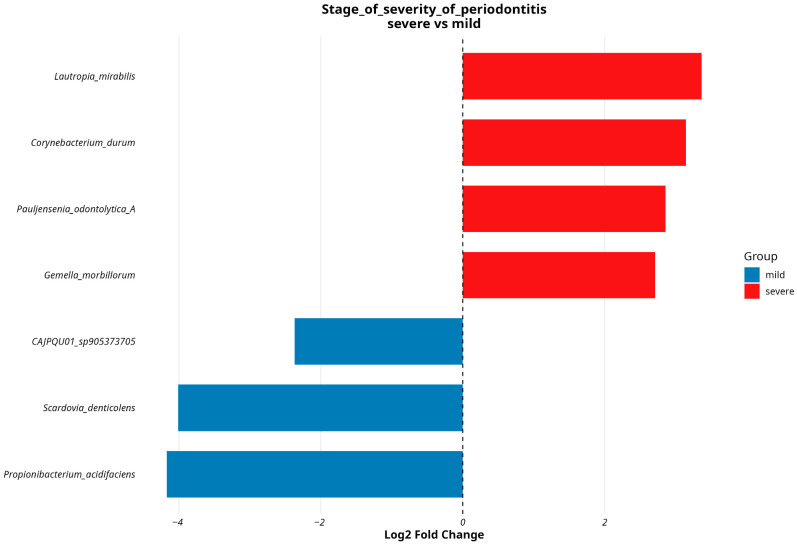
DESeq2 results for differentially abundant MAGs testing associations with stage of severity of periodontitis. Species associated with a milder course of disease are colored in blue; species associated with a more severe course of periodontitis are colored in red.

Our findings were additionally verified by estimating the prevalence of these species and calculating their log10 abundance. We conducted a similar analysis for the MAGs dataset using the same covariates as a grouping factor. We observed four MAGs displaying abundance changes linked with severe stage of periodontitis: *Lautropia mirabilis*, *Gemella morbillorum*, *Corynebacterium durum*, and *Pauljensenia odontolytica*; and three MAGs showing associations with mild stage of periodontitis: *CAJPQU01_sp905373705*, *Scardovia denticolens* and *Propionibacteruim acidifaciens* (Figure 11). Info about BH corrected *p*-adjusted values and logFC values of differentially abundant MAGs in regard to their associations with stage of severity of periodontitis shown on the plot is presented in Supplementary Table S2.

**Figure 12:**
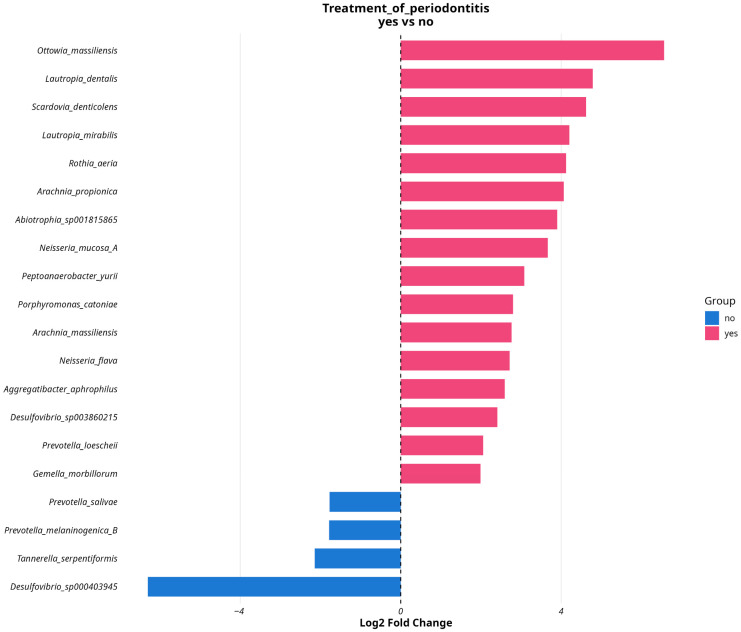
DESeq2 results for differentially abundant MAGs testing associations with antibiotics treatment. Species associated with prior treatment are colored in light-blue; species associated with absence of prior treatment are colored in pink.

16 MAGs showed abundance changes linked with prior treatment of periodontitis, namely *Ottowia massiliensis*, *L. mirabilis*, *L. dentalis*, *S. denticolens*, *G. morbillorum*, *Peptoanaerobacter yurii*, *Porphyromonas catoniae*, *Rothia aeria*, *Arachnia propionica*, *Abiotrophia_sp001815685*, *Neisseria mucosa*, *Arachnia massiliensis*, *Neisseria flava*, *Aggregatibacter aphrophilus*, *Desulfovibrio_sp_003860215* and *Prevotella loescheii*, and 4 MAGs were associated with absence of prior treatment of periodontitis: *Prevotella melaninogenica*, *Prevotella salivae*, *Desulfovibrio_sp000403945* and *T. serpentiformis*. (Figure 12). Info about BH corrected *p*-adjusted values and logFC values of differentially abundant MAGs in regard to their associations with treatment of antibiotics shown on the plot is presented in Supplementary Table S3.

### 3.5. Analysis of Microbial Functional Composition in Relation to Stage of Severity and Treatment of Periodontitis

**Figure 13:**
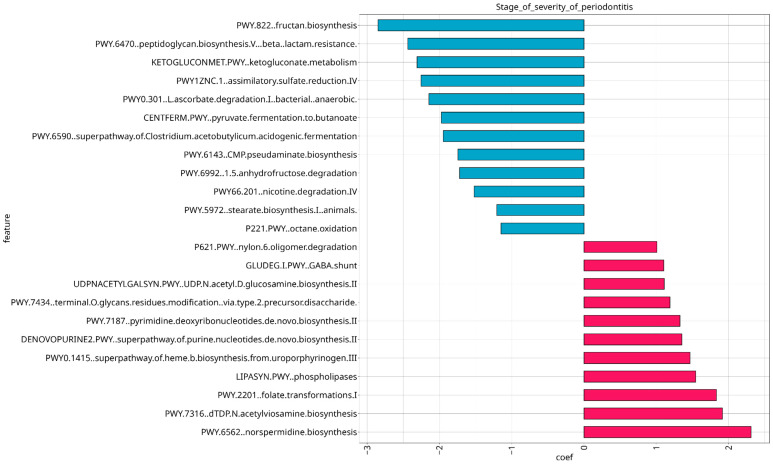
Significant microbial pathways associated with stage of severity of periodontitis revealed by MaAsLin2 analysis. Pathways exhibited negative correlation are colored in blue, pathways revealed positive correlation are colored in red.

We conducted an analysis of predicted joint metabolic pathways using MaAsLin2 to reveal associations between functional microbial features and metadata covariates of interest, specifically the stage of severity of periodontitis and treatment of periodontitis. As a result, 23 metabolic pathways showed their association with the severe stage of periodontitis, 7 of them exhibited negative correlations, and 16 of them demonstrated positive correlations; q-values and coef values of them are stored in Supplementary Table S4 (Figure 13).

**Figure 14:**
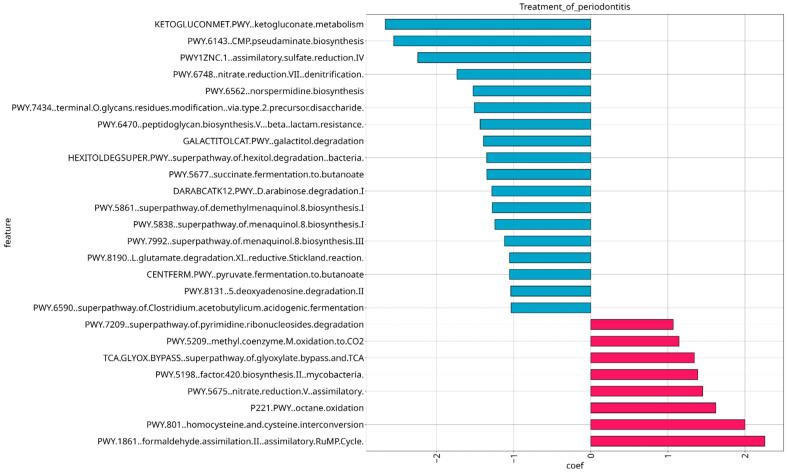
Significant microbial pathways associated with treatment of periodontitis revealed by MaAsLin2 analysis. Pathways that exhibited negative correlation are colored in blue and pathways that revealed positive correlation are colored in red.

Among different pathways related to carbohydrates and polysaccharides metabolism, amino acid metabolism and cofactors metabolism with varying correlations, negative correlation was observed for pathway PWY-6470: peptidoglycan biosynthesis V (beta-lactam resistance) (coef = −1.062), suggesting the presence of antibiotic-resistant bacteria which may hinder treatment effectiveness. Synthesis of different polysaccharides which are components of lipopolysaccharides and polyamines are positively correlated with stage of severity of periodontitis, notably UDPNACETYLSYN.PWY: N-acetyl-D-glucosamine biosynthesis (coef = 1.356), PWY-7332: superpathway of UDP-N-acetylglucosamine-derived O-antigen building blocks biosynthesis (coef = 1.404) and PWY-6562: norspermidine biosynthesis (coef = 2.219). PWY-7820: teichuronic acid biosynthesis, which is a component of the Gram-negative bacteria cell wall, is also positively correlated (coef = 1.845), suggesting active bacterial growth. A total of 27 metabolic pathways showed associations with treatment of periodontitis, 9 of them revealed positive correlation with treatment (including pathways for cofactor biosynthesis), and 18 showed negative correlation with treatment of periodontitis, demonstrating decreased metabolic activity; their q-values and coef values are stored in Supplementary Table S5. It should be noted that the aforementioned pathway PWY-6470: peptidoglycan biosynthesis V (beta-lactam resistance) also exhibits negative correlation in association with treatment of periodontitis. (Figure 14).

### 3.6. The Periodontal Microbiota’s Network Structure Shows Variations Across Patients with Differing Severity Levels

**Figure 15:**
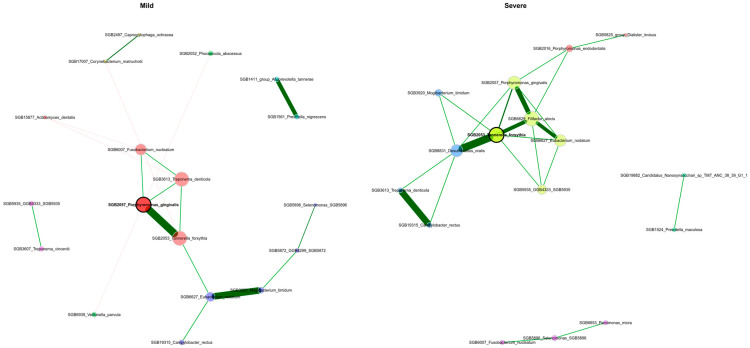
Bacterial co-occurrence networks between patient groups with mild and severe periodontitis, inferred using the SPIEC-EASI method. Hub taxa (keystone nodes) were identified as those with eigenvector or degree centrality values exceeding the 95th percentile, and their size is scaled accordingly. Node colors represent clusters defined by the Louvain algorithm, with a shared color between networks indicating a cluster containing at least two same taxa in both networks. A single layout is used for both networks, from which nodes that were unconnected in both groups or those that exhibited an edge selection frequency below a stability threshold of 0.8 were removed.

**Figure 16:**
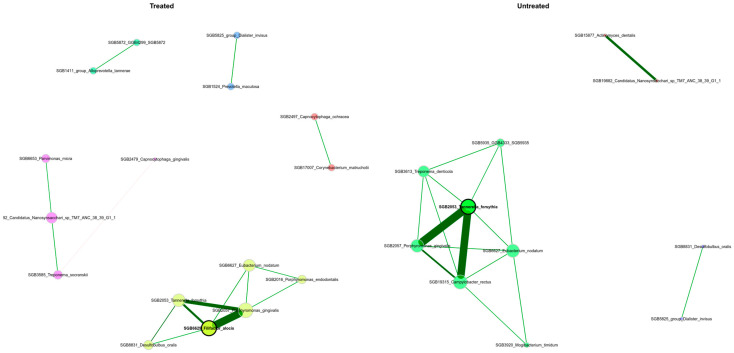
Bacterial co-occurrence networks between patient groups who received or did not receive treatment of periodontitis, inferred using the SPIEC-EASI method. Hub taxa (keystone nodes) were identified as those with eigenvector or degree centrality values exceeding the 95th percentile, and their size is scaled accordingly. Node colors represent clusters defined by the Louvain algorithm, with a shared color between networks indicating a cluster containing at least two of the same taxa in both networks. A single layout is used for both networks, from which nodes that were unconnected in both groups or those that exhibited an edge selection frequency below a stability threshold of 0.8 have been removed.

We utilized SPIEC-EASI to investigate bacterial interactions and evaluate potential variations in the organization of microbial communities between the mild and severe groups. The robust network analysis with filtering of edges by stability selection and using optimal taxa-to-sample ratio (32 core species on 28 samples) revealed distinct characteristics in the microbial communities of the study cohort both in regard to stage of severity of periodontitis and treatment status (Figure 15 and Figure 16). Edges stability selection frequencies are reported in Supplementary Files S5 and S6.

## 4. Discussion

It should be addressed that ‘oral microbiome’ is an umbrella term and comprises different distinct microbial communities from and within their specific niches, such as saliva metagenome, dental plaque metagenome, periodontal pockets metagenome etc., thus making oral microbiome a heterogenous mixture. This paper compares the microbial composition of periodontal pockets of individuals with mild periodontitis cases to those with more severe cases, focusing on the specific niche, rather than comparing healthy individuals to subjects suffering with periodontitis. In this vein, we can justify the absence of a group of healthy patients by the absence of these periodontal pockets in the healthy oral cavity. A similar approach to study cohort formation was also used in [32], where researchers investigated connections between taxonomic and functional composition of periodontal microbiota and pocket depth. The selected study cohort and scope of conducted research is especially relevant to the more global aim of periodontitis treatment and mitigation. However, including a control group of healthy individuals may be useful for direct comparison of microbiome, taxonomic, and functional composition of healthy and pathogenic microbiomes for future large-scale studies.

In our study, we utilized the WGS metagenomic analysis to compare the taxonomic composition of the periodontal microbiota in patients with different stages of periodontitis upon their visit to the dentist. Combining the results of the CT scans and RT-PCR testing for *P.gingivalis*, we successfully categorized patients according to disease severity. Although we did not find any significant differences in alpha and beta-diversity between different stages of periodontitis, we were able to find a dissimilarity in alpha-diversity between different BMI groups, suggesting that as a risk factor. Identifying and understanding these risk factors can be crucial for managing the outcomes of periodontitis, thus enabling us to adjust preventive measures and treatments, potentially mitigating the severity and spread of the disease. We found no beta-diversity differences between patients of mild and severe groups; however, statistically significant differences were observed between sexes (which may be detected because of gender imbalance and subsequent shifts in statistics), as well between patients who underwent antibiotics treatment in the last six months prior to their visit to the dentist, thus providing a long-lasting effect on periodontal microbiome composition. Various factors can contribute to these results and ambiguities, such as limitations in study design, sample size, patient demographics, methodologies, and geographic locations. PERMANOVA analysis revealed that covariate “cardiovascular diseases” has a statistically significant impact on microbial taxonomic composition, explaining the variance between patients. There are some reports demonstrating a connection between this group of illnesses with periodontitis [33]; however, exact mechanisms involved in linkage between periodontal disease and these maladies are not clearly identified at the time.

Obtained results of DESeq analysis showed the complex structure of the oral microbiome, where some organisms can be present in both a healthy and a periodontal microbiome, thus drawing conclusions about disease progression key factors and their impact more difficult. We observed some statistically significant differences in the abundance of two species in the periodontal microbiota between patients who underwent treatment of periodontitis. Not much information is available about *P.enoeca*, which was first isolated from gingival crevice [34], and according to some studies, e.g., [35] this species has a higher abundance in metagenomes of patients that suffered from both Crohn’s disease and periodontitis, but its impact on disease dynamics is yet to be discovered.

We found no taxons associated with the mild stage of periodontitis, and five taxons which are associated with the severe stage of periodontitis. One of them is *A. segnis*, which was reclassified from *Haemophilus* genus [36], is a part of HACEK group of microorganisms [37], and was linked with skin and soft tissue infections [38], but its role in periodontitis development is not defined.

A similar DESeq analysis for MAGs dataset also yielded ambiguous results. We found four MAGs showing association with a severe stage of periodontitis. *L. mirabilis* is a bacterial species present in both healthy individuals and periodontitis patients, with evidence indicating its preferential association with periodontal health [39,40]. However, this bacteria and its impact in microbiome composition is poorly understood. *C. durum*, a prolific biofilm and extracellular matrix producer is a highly abundant member of oral microbiota and can play crucial roles in maintaining oral health through mechanisms involving hydrogen peroxide production and membrane vesicle secretion, which can inhibit pathogenic species and modulate host immune responses [41]. We can suggest that high abundance of *C. durum* in patients with severe periodontitis could be a mechanism to slow down ongoing infection, although its effectiveness may be limited. The role of *G. morbillorum* is currently poorly understood, although its abundance is higher in healthy oral flora compared to those in patients with periodontal disease. It has been shown that other *Gemella spp.* may inhibit growth of *P. gingivalis*, thus slowing the speed of disease progression [42]. *Pauljensenia odontolytica*, specified in NCBI as *S. odontolytica*, may play a role of putative pathogenic bacteria, according to [43]. The association of three MAGs with a mild stage of periodontitis was detected. Although at the moment the link between *P. acidifaciens* and periodontitis has not been found, it is known that *P. acidifaciens* is highly present in patients with active caries [44], so we may suggest that such bacteria could also play a role in periodontitis progression. *S. deticolens*, formerly known as *Bifidobacterium denticolens*, was isolated from human caries [45], but its role and impact on oral health is yet to be studied.

Four MAGs were associated with the absence of treatment of periodontitis. *P. melaninogenica* is one of the predominant species in the oral microbiome and was frequently found in both healthy patients [46] and patients with periodontitis [47]. However, *P. melaninogenica* can be a participant in periodontitis progression via stimulation of expression of virulence factor via regulation by proteases and cell surface proteins secreted by *P. gingivalis* and *T. forsythia* [48]. *Prevotella* spp. including *P. salivae* are commonly associated with human infections such as dental caries and periodontitis, due to the production of various virulence factors that cause infection and proliferation in host tissues [49]. Data on *T. serpentiformis* demonstrates the potential for interfering with biological processes driving periodontal inflammation, albeit being a close relative of pathogenic *T. forsythia* [50]. In contrast, 16 MAGs were associated with the treatment of periodontitis. *O. massiliensis*, a recently characterized species first isolated from human fecal samples in 2022 [51] and later detected in oral samples, demonstrated pathogenic potential based on clinical case reports [52]. *L. dentalis* is a novel species isolated from gingivitis lesion [53] and its direct impact on oral health has not been studied. *P. catoniae*, together with *C. durum,* are core members to both the dental and periodontal health conditions [54]. *P. yurii*, formerly known as *E. yurii*, is overrepresented in the oral microbiome of patients with aggressive periodontitis, but its role in the dynamics of the disease is yet to be discovered [55]. *Neisseria* spp., are common commensal members of oral microbiome, detectable in both healthy and dysbiotic communities, and probably associated with a healthy status of oral microbiome [56], but their impact remain largely unknown. *R. aeria* along with closely related bacteria from the same genus are strongly associated with periodontal health [57], due to nitrate-reducing activity that may decrease periodontal inflammation [58]. *A. propionica*, later reclassified as *Propionibacterium propionicum*, was found in periodontic lesions, and some studies report that Propionibacterium genus are associated with primary and secondary endodontic infections [59]. *A. massiliensis*, also known as *Pseudopropionibacterium massiliense* [60], is a novel species isolated from oral microbiota in 2019, so its impact remains unknown for the present moment. As discussed earlier, bacterial species of *Aggregetibacter* genus (*A. aphrophilus* in our case) are members of HACEK group and often found in oral microbiome composition; due to their adhesive properties and presence of different virulence factors, they can accelerate progression of periodontal damage [61]. The compromised periodontal epithelial barrier in periodontitis may facilitate bacterial translocation into the systemic circulation, thereby establishing a potential mechanistic link between periodontal disease and severe systemic complications, e.g., endocarditis [62]. *P. loeschii* are also potent in biofilm formation [63,64], but its role in periodontitis progression is not described.

In conclusion, the obtained results showed some ambiguity, where both pathogenic and non-pathogenic bacteria co-exist, displaying complex interactions within the host’ periodontal pockets. It should be noted that analysis revealed potential associations for novel and poorly studied bacteria, promoting future research in the microbiology and genomics of such species. Observed statistical significance of findings should not replace thorough biological interpretation, though, and these findings have to be verified using wet lab methods to be considered absolutely true. Conducted analysis may pave the way for future investigations, narrowing the focus from broader context to more precise searches.

The metabolic processes within the periodontal microbiome are crucial in determining the composition of periodontal microbiota, its function, its and influence on the host. Analysis of metabolic pathways showed that pathways related to lipopolysaccharide metabolism and polyamine biosynthesis are positively correlated with the severe stage of periodontitis. According to other reports, lipopolysaccharides from periodontal bacteria may act as potent immunostimulatory molecules and may also act as a pyrogens, speeding inflammation and tissue damage within the periodontal pocket [65,66]. Likewise, pathways involved in polyamine biosynthesis, responsible for generating bioactive compounds like norspermidine, are observed at higher levels in areas affected by periodontitis [67] and may also increase bacterial resistance to antibiotics [68], potentially making treatment more complicated. However, these computational findings require additional verification through controlled wet-lab experiments to confirm any potential causal relationships.

The network analysis highlighted differences in the microbiome structure between the mild and severe groups of periodontitis and relation with treatment of periodontitis. Both network analyses revealed a stable hub of red complex bacteria (*P. gingivalis*, *T. forsythia)* interacting with *F. alocis,* a novel periodontal pathogen playing a significant role in disease dynamics [69], along with other bacteria, e.g., *Desulfobulbus oralis*, a novel pathobiont of human periodontal microbiome which can trigger a proinflammatory response in oral epithelial cells, suggesting a direct role in the development of periodontal disease [70]. Networks for mild and severe stages of periodontitis also reveal interaction with *Campylobacter rectus*, a member of the orange complex bacteria which also play a pathogenic role in periodontitis [71]. Presence of *C. gingivalis*, a member of the green complex of bacteria which is involved in early biofilm colonization and associated with periodontitis [72], interacting with *T. sockranskii*, a bacteria which is also associated with periodontal inflammation [73], was also detected. Network analysis also found an interaction with *M. timidum*, a novel discovered species and is periodontitis-associated bacteria, as its levels are substantially higher in inflammatory conditions than in healthy microbiome [74] and also with *Eubacterium nodatum* and *Treponema denticola*, which according to [75] have a strong association with periodontitis. To sum up, the resulting networks of interactions between periodontal microbiome bacteria and configuration of such networks allow us to estimate structure and interactions within the microbiome, unveiling complex mechanisms of periodontal damage. Although the presence and interactions of pathogenic bacteria were detected even after periodontitis treatment was conducted, it does not allow us to suggest that the treatment measures were insufficient to eliminate pathogenic bacteria without using other methods such as estimation of inflammatory markers or other clinical analyses.

In summary, our findings demonstrate that the stage of severity of periodontitis has different effects on the taxonomic and functional composition of the periodontal microbial community in both mild and severe groups of patients, suggesting differential microbial responses and in return influences on microbiome composition of periodontitis dynamics. According to the obtained results, different covariates and features such as cardiovascular diseases and antibiotics treatment in the last six months prior to hospital admission may have an influence on oral microbiome diversity, which corroborates prior research data. The results of differential abundance testing revealed co-existing healthy and pathogenic flora inside periodontal pockets. The results of network analysis allow us to detect complex interactions between the pathogenic bacteria of the periodontal microbiome. The obtained results lay the groundwork for future prospective large-scale studies on bigger cohorts to validate the oral microbiome’s role as a predictive biomarker for periodontitis severity and treatment effectiveness.

## 5. Limitations of This Study

Findings from the present study should be interpreted in light of its limitations, which include a relatively small study cohort. The observed gender imbalance—with the mild group having a higher proportion of men and the severe group being predominantly women—may introduce confounding effects that limit the generalizability of our findings, as gender-related differences could influence the outcomes. Since antibiotics can be obtained without a doctor’s prescription in Russia, and our knowledge of antibiotic usage relies on patients’ self-disclosures, obtaining accurate information about the particular antibiotics that were used poses a challenge. Nevertheless, we cannot ignore this information, as it may have a significant impact on the microbiome. While the study provides insights into how the periodontal microbiota composition correlates with the severity of periodontitis, it stops short of establishing a direct causal relationship between specific microbiota changes and periodontitis outcomes because of vertical analysis.

Despite these limitations, our multi-approach exploratory analysis of samples from a fully characterized group of 28 patients with mild and severe stages of periodontitis are in line with previous findings and yielded some thought-provoking conclusions. Unfortunately, the study cohort size was relatively small, so outcomes are mostly exploratory and should be validated in larger cohorts before considered fully reliable.

## Figures and Tables

**Table 1:** Descriptive statistics of quantitative metadata variables.

Sex	Variable	Mean	Min	Max	SD
Male	Weight	90.21	58	110	17.75
	Height	178.36	168	188	6.68
	BMI	26.56	20.55	33.90	4.53
Female	Weight	69.08	53	89	10.66
	Height	169.15	150	185	8.93
	BMI	24.12	19.71	29.98	3.82

## Data Availability

Sequencing reads for WGS sequencing were deposited to NCBI BioProject under project name PRJNA1265940. Code for data analysis is deposited in Github repository: https://github.com/META-SBM/WGS_periodontitis (accessed 30 August 2025).

## Supplementary Materials

The following supporting information can be downloaded at: https://www.mdpi.com/article/10.3390/dj13120590/s1, Supplementary Table S1: Questionnaire sample sheet template; Supplementary Table S2: DESeq results table for BH corrected p-adjusted values and logFC values of differentially abundant MAGs in regard to their associations with the stage of severity of periodontitis shown on Figure 12; Supplementary Table S3: DESeq results table for BH corrected p-adjusted values and logFC values of differentially abundant MAGs in regard to their associations with treatment of antibiotics shown on Figure 13; Supplementary Table S4: Results of MaAsLin2 analysis of predicted joint metabolic pathways and their associations with the stage of severity of periodontitis; Supplementary Table S5: Results of MaAsLin2 analysis of predicted joint metabolic pathways and their associations with treatment of periodontitis; Figure S1: Barplot for read counts of each sample, displaying both the total reads number, as well as number of human and bacterial reads. The 10 million reads mark was used as a cutoff for excluding some samples; Figure S2: Top 15 abundant genera present in WGS dataset, samples are grouped by stage of severity of periodontitis; Figure S3: Top 15 abundant species present in WGS dataset, samples are grouped by stage of severity of periodontitis; Figure S4: Heatmap showing the abundance of 143 MAGs in patients with mild and severe periodontitis, MAGs reads abundance is CLR normalized and Ward.D2 clustered for better visualization of patterns; Figure S5: Alpha diversity by Shannon index. Box plots illustrate alpha diversity by Shannon index in bacterial MAGs of 28 patient samples across different variables. Median values and interquartile ranges have been indicated in the plots; Figure S6: Composed Shannon index alpha-diversity (A) and PCoA plots (B) of beta-diversity estimated with Bray–Curtis distance and statistical test results (ANOSIM test) between MAGs composition of different variables of interest. ‘A’ for all subplots is the alpha-diversity plot of the respective variable, ‘B’ for all subplots is the beta-diversity plot of the respective variable. Median values and interquartile ranges have been indicated in the plots. Violin plots are displaying statistical differences for each PCoA component. Ellipses are drawn around 95% confidence level of samples based on a multivariate t-distribution; Figure S7: Composed Shannon index alpha-diversity (A) and PCoA plots (B) of beta-diversity estimated with Bray–Curtis distance and statistical test results (ANOSIM test) between WGS taxonomic composition of different variables of interest. ‘A’ for all subplots is the alpha-diversity plot of the respective variable, ‘B’ for all subplots is the beta-diversity plot of the respective variable. Median values and interquartile ranges have been indicated in the plots. Violin plots are displaying statistical differences for each PCoA component. Ellipses are drawn around 95% confidence level of samples based on a multivariate t-distribution; Figure S8: Composed Shannon index alpha-diversity (A) and PCoA plots (B) of beta-diversity estimated with Bray–Curtis distance and statistical test results (ANOSIM test) between metabolic pathways composition of different variables of interest. ‘A’ for all subplots is the alpha-diversity plot of the respective variable, ‘B’ for all subplots is the beta-diversity plot of the respective variable. Median values and interquartile ranges have been indicated in the plots. Violin plots are displaying statistical differences for each PCoA component. Ellipses are drawn around 95% confidence level of samples based on a multivariate t-distribution; Supplementary FIle S1: Results of correlation analysis on metadata covariates and VIF analysis to exclude multicollinear covariates; Supplementary Files S2–S4: Results of PERMDISP test analysis of metadata distribution of covariates of interest for each of dataset, respectively (WGS/MAGs/metabolic pathways); Supplementary File S5: Edge stability selection frequencies for bacterial networks in relation of mild and severe stage of periodontitis; Supplementary File S6: Edge stability selection frequencies for networks in relation of treatment of periodontitis.
