# Supplementary material for: In-Depth Multi-Approach Analysis of WGS Metagenomics Data Reveals Signatures Potentially Explaining Features in Periodontitis Stage Severity
Source: Dent J (Basel). 2025 Dec 8;13(12):590. doi: 10.3390/dj13120590 (PMC12731556; doi:10.3390/dj13120590)
Supplement: Supplementary file 1 [file dentistry-13-00590-s001.zip › WGS Periodontitis Suppl Figs only.pdf]

Supplementary Figures for Article: In-depth analysis of WGS metagenomics data unveils signatures explaining features in periodontitis stage severity

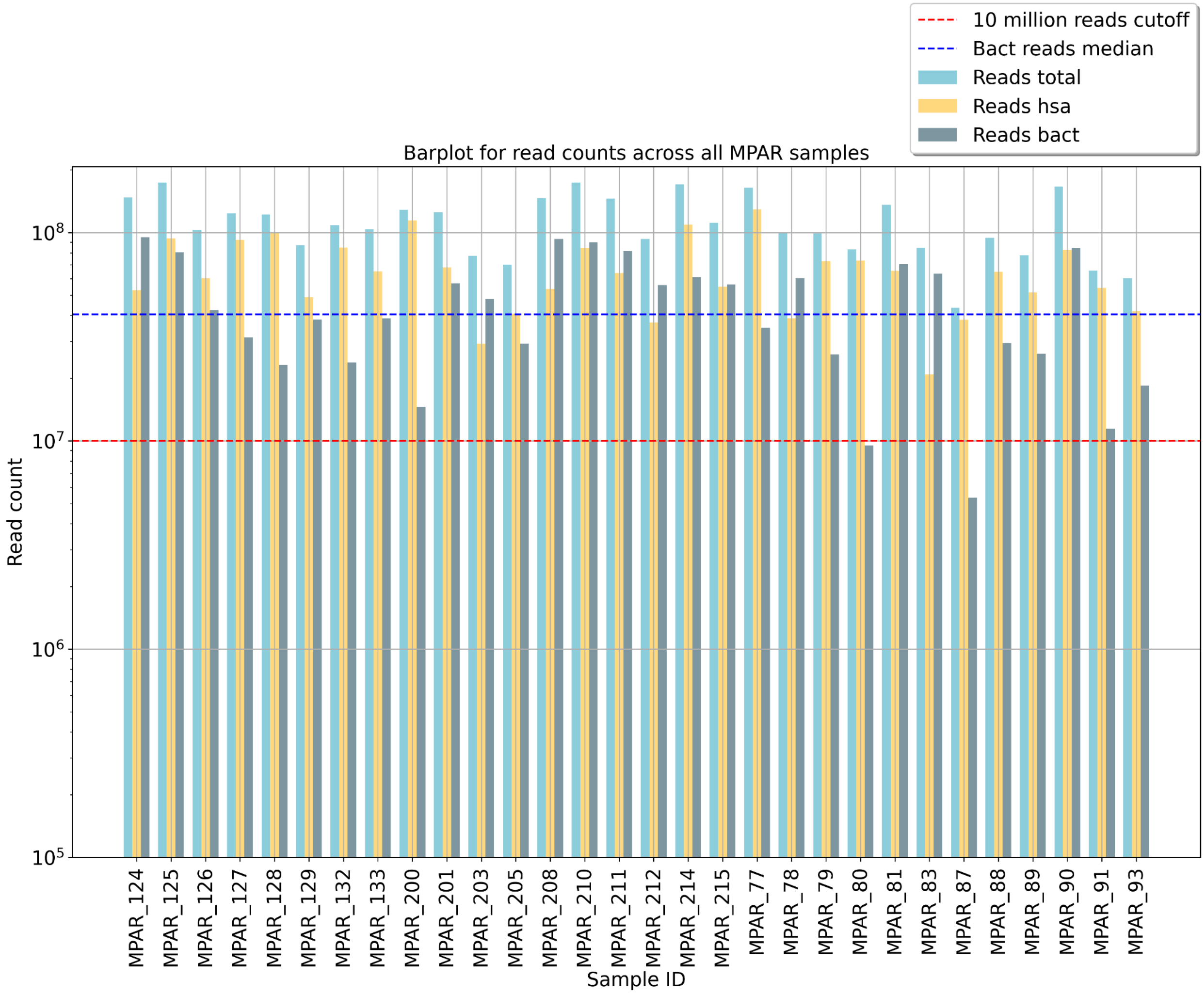

Figure S1. Barplot for read counts of each sample, displaying both total reads number, as well as reads of human and bacterial reads. The 10 million reads mark was used as a cutoff for excluding some samples.

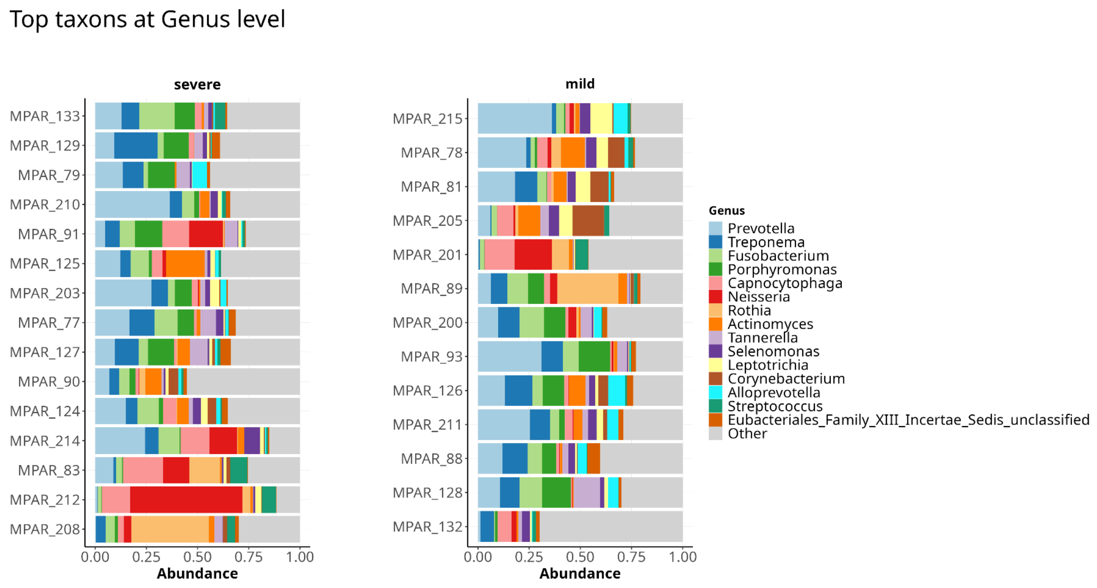

Figure S2. Top 15 abundant genera present in WGS dataset. Samples are grouped by stage of severity of periodontitis

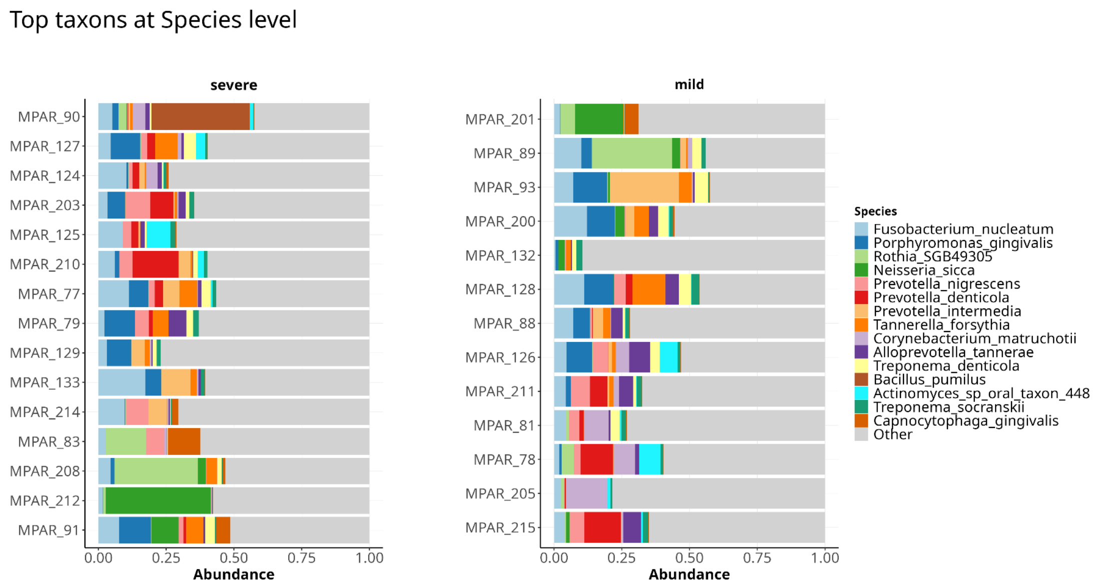

Figure S3. Top 15 abundant species present in WGS dataset. Samples are grouped by stage of severity of periodontitis.

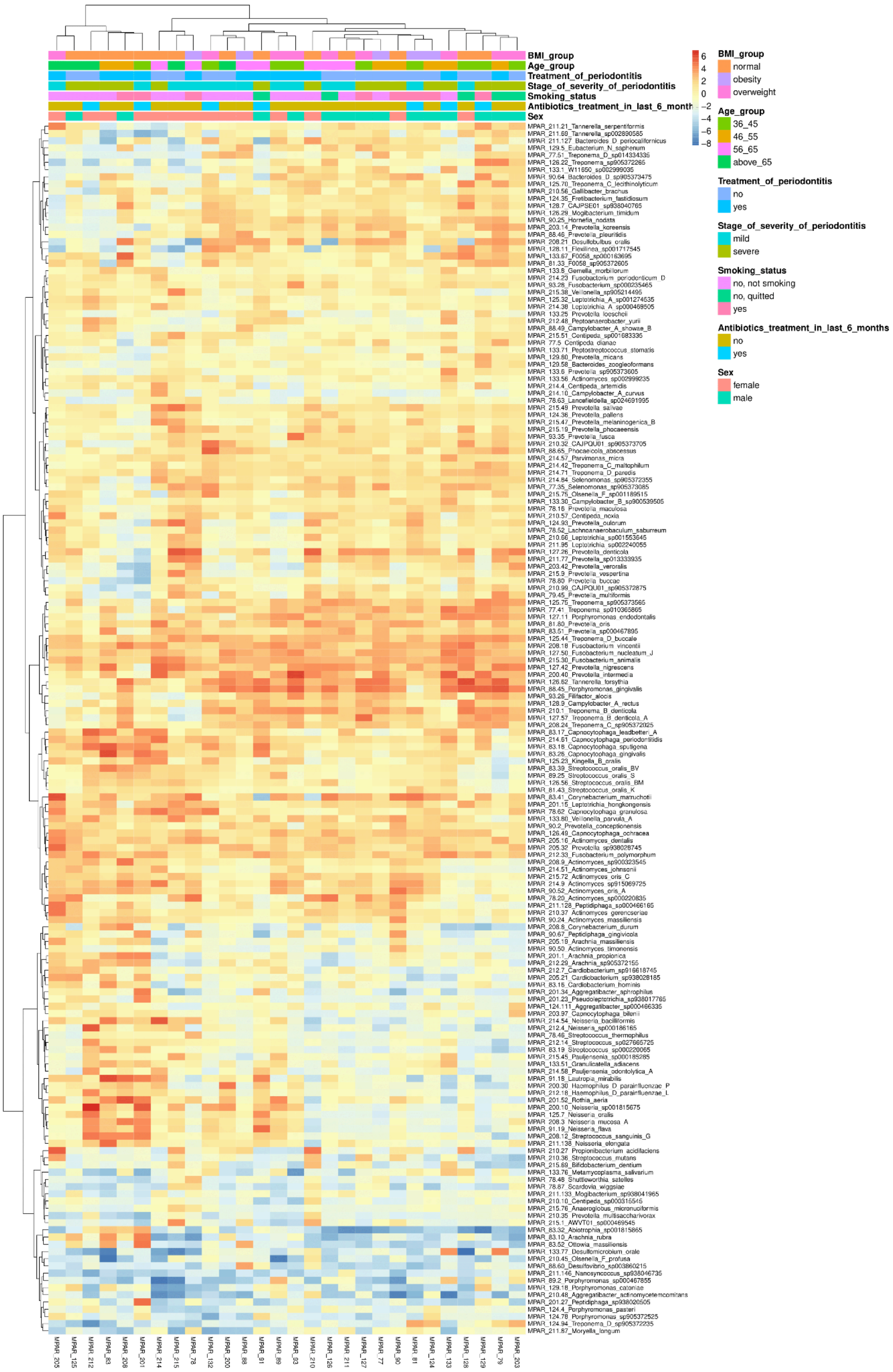

Figure S4. Heatmap showing the abundance of 168 MAGs with prevalence filter = 0.1 in patients with mild and severe periodontitis. MAGs read abundance is CLR normalized and Ward.D2 clustered.

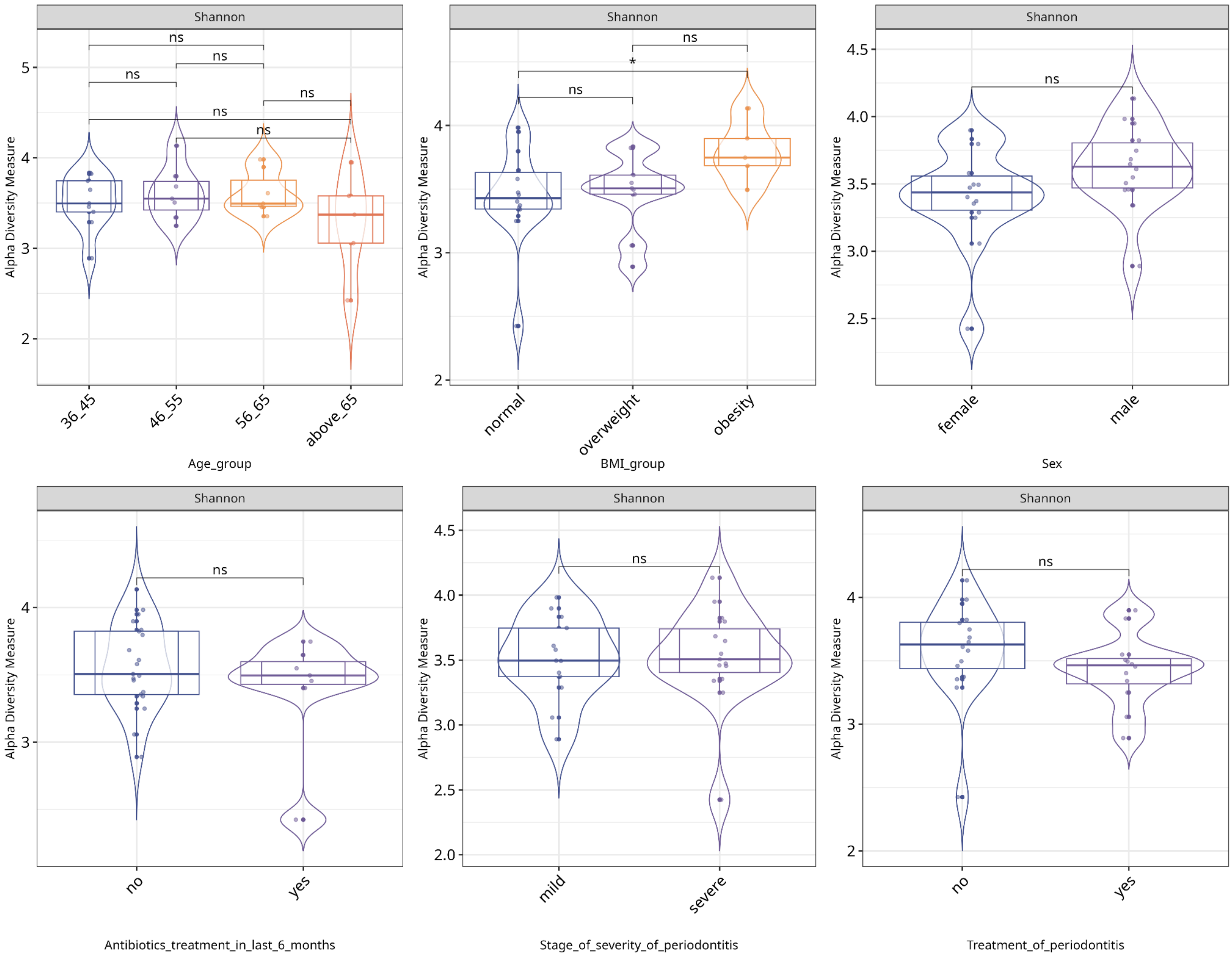

Figure S5. Alpha diversity by Shannon index. Box plots illustrate alpha diversity by Shannon index in bacterial MAGs of 28 patient samples across different variables. Median values and interquartile ranges have been indicated in the plots.

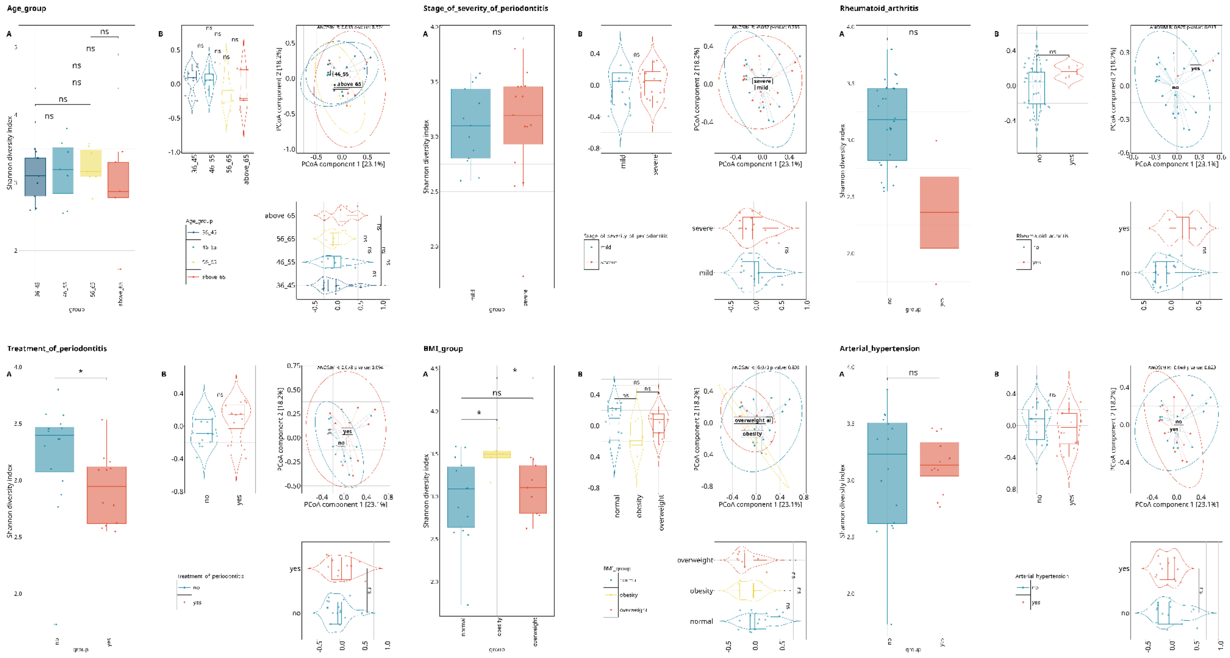

Figure S6. Composed Shannon index alpha-diversity and PCoA plots of beta-diversity estimated with Bray-Curtiss distance and statistical test results (ANOSIM test) between MAGs composition of different variables of interest. ‘A’ for all subplots is the alpha-diversity plot of the respective variable, ‘B’ for all subplots is the beta-diversity plot of the respective variable.

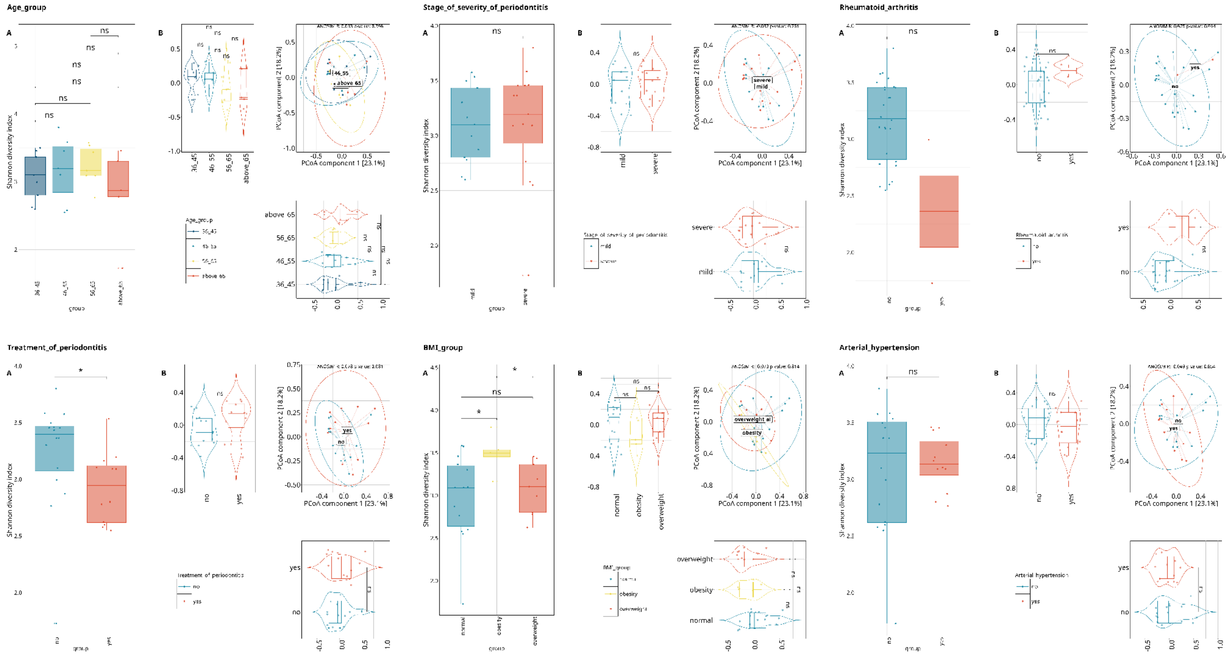

Figure S7. Composed Shannon index alpha-diversity and PCoA plots of beta-diversity estimated with Bray-Curtiss distance and statistical test results (ANOSIM test) between WGS taxonomic composition of different variables of interest. 'A' for all subplots is the alpha-diversity plot of the respective variable, 'B' for all subplots is the beta-diversity plot of the respective variable.

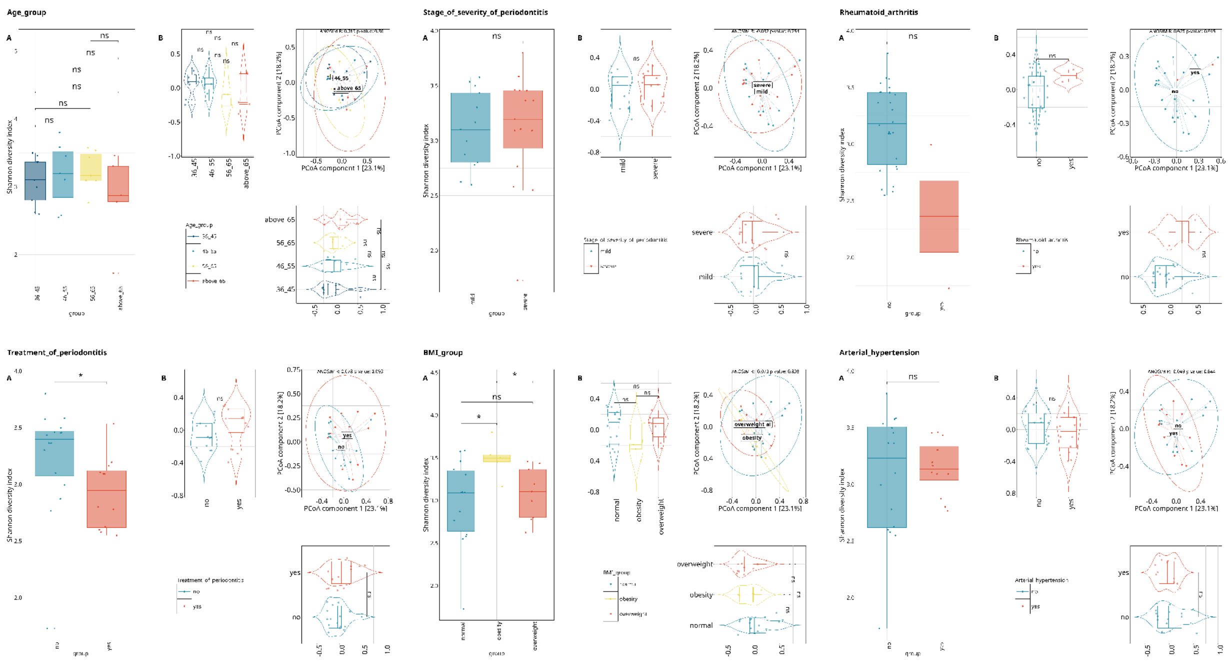

Figure S8. Composed Shannon index alpha-diversity and PCoA plots of beta-diversity estimated with Bray-Curtiss distance and statistical test results (ANOSIM test) between metabolic pathways composition of different variables of interest. ‘A’ for all subplots is the alpha-diversity plot of the respective variable, ‘B’ for all subplots is the beta-diversity plot of the respective variable.
