# Supplementary material for: In-Depth Multi-Approach Analysis of WGS Metagenomics Data Reveals Signatures Potentially Explaining Features in Periodontitis Stage Severity
Source: Dent J (Basel). 2025 Dec 8;13(12):590. doi: 10.3390/dj13120590 (PMC12731556; doi:10.3390/dj13120590)
Supplement: Supplementary file 1 [file dentistry-13-00590-s001.zip › WGS_Periodontitis_Suppl_Tables _rev1.pdf]

**Serial No. Of study participant:MPAR_**					
**1**	**Date of sample collection**				
**2**	**Age (years)**				
**3**	**Weight / height**				
**4**	**Sex**				
**5**	**Medical history**			**Yes**	**No**
5.1	Arterial hypertension				
5.2	Cardiovascular diseases				
5.3	Chronic bronchitis				
5.4	Bronchial asthma				
5.5	Rheumatoid arthritis				
5.6	Tuberculosis				
5.7	Kidney diseases				
5.8	Diabetes melitus				
5.9	Oncology disorders				
5.1	IBD (Inflammatory bowel disease)				
**6**	**Antibiotics treatment in last 6 months**				
6.1	If Yes, please specify date of last dose administration				
6.2	Specify a name of antibiotic				
**7**	**Please specify other medications administered to patient:**				
**8**	**Smoking status**				
8.1	No, not smoking	8.2	No, quit smoking	8.3	Yes
**9**	**How long ago did you quit smoking?**				
9.1	Less than 1 year ago	9.2	1-5 years ago		
9.3	5-10 years ago	9.4	More than 10 years ago	9.5	Not sure
**10**	**Stage of severity of periodontitis**				
10.1	Light	10.2	Mild	10.3	Severe
**11**	**Treatment of periodontitis**				
11.1	No	11.2	Yes		
11.3	Last course of treatment months ago				
**12**	**Patient category**				
12.1	First time applicant	12.2	Previously observed		

baseMean	log2FoldChange	lfcSE	stat	pvalue	padj	Species	group
29980.276005791	-4.17003104240264	0.884308667062815	-4.71558314163445	2.41019446998656E-06	0.000172328904604039	Propionibacterium_acidifaciens	mild
198.299877946752	-4.00989609823857	1.12080383100258	-3.57769663818122	0.000346635427055512	0.0123922165172345	Scardovia_denticolens	mild
95588.6768193708	-2.37165718131369	0.749817601930167	-3.1629788033898	0.00156163701522525	0.0319020133110301	CAJPQU01_sp905373705	mild
19949.8396471992	2.7105767258179	0.540870995247555	5.0115032043404	5.40064840435536E-07	7.72292721822817E-05	Gemella_morbillorum	severe
15139.4055885629	2.85647826775885	0.79244350487497	3.60464594660226	0.000312578757200459	0.0123922165172345	Pauljensenia_odontolytica_A	severe
15528.1228052453	3.14317809238801	0.959699445540925	3.27516922823311	0.00105598695636652	0.0273274118423724	Corynebacterium_durum	severe
113540.037848301	3.36430010582456	1.03458346828671	3.2518401936152	0.00114660469268695	0.0273274118423724	Lautropia_mirabilis	severe

baseMean	log2FoldChange	lfcSE	stat	pvalue	padj	Species	group
11784.140718877	-6.30455282117014	1.19058349279411	-5.29534707924963	1.18790567338969E-07	8.49352556473632E-06	Desulfovibrio_sp000403945	no
31718.8493612168	-2.14566293890838	0.688857797012267	-3.1148125900797	0.00184061814270229	0.0164767719544923	Tannerella_serpentiformis	no
29561.0731705772	-1.78801572587627	0.648858831565644	-2.75563133133587	0.00585789822908458	0.0418839723379548	Prevotella_melaninogenica_B	no
46712.5850048548	-1.77522464039998	0.570015907541804	-3.11434227871929	0.00184355490399914	0.0164767719544923	Prevotella_salivae	no
19281.9501594093	1.98928823970266	0.597609243959285	3.32874409124466	0.000872385227580069	0.0103959239619958	Gemella_morbillorum	yes
16033.2265727312	2.05677176152272	0.739188209491239	2.78247371253167	0.00539462353287806	0.0406016402737665	Prevotella_loescheii	yes
1114.65373724629	2.4098259659336	0.76004738192205	3.17062596786992	0.00152110864720975	0.0164767719544923	Desulfovibrio_sp003860215	yes
8518.96273113201	2.59259693901223	0.863872919901941	3.00113231852032	0.00268977656486113	0.0226257675750083	Aggregatibacter_aphrophilus	yes
44815.0146481714	2.71745828866085	0.912276125629166	2.97876729678387	0.00289410479576417	0.0229920547663487	Neisseria_flava	yes
25885.0120525742	2.76494040956035	0.885716149402102	3.12170034545132	0.00179809856072944	0.0164767719544923	Arachnia_massiliensis	yes
1560.05210586865	2.79827069428913	0.813265960567725	3.44078177369641	0.000580036141449404	0.00754046983884225	Porphyromonas_catoniae	yes
23059.3879909355	3.08041001122487	0.620253962742903	4.96636893314248	6.82181205930802E-07	3.25173041493682E-05	Peptoanaerobacter_yurii	yes
81633.7045368594	3.66400203373631	0.892121149851189	4.10706778372812	4.00713662583515E-05	0.000818600767849181	Neisseria_mucosa_A	yes
3753.29120686536	3.89981659786744	1.06780914223944	3.65216633160551	0.000260037358715265	0.00371853422962829	Abiotrophia_sp001815865	yes
75758.3365387898	4.06427952461516	0.917073500687683	4.43179256795393	9.34529001287432E-06	0.000334094117960257	Arachnia_propionica	yes
226983.29234817	4.12282173247676	0.949248844162241	4.34324651310517	1.40392448344459E-05	0.000401522402265152	Rothia_aeria	yes
301849.355307196	4.20071804835917	1.08212713303703	3.88190806801942	0.000103640059886901	0.00164672539598076	Lautropia_mirabilis	yes
289.993950859281	4.62027231771837	1.1399225449473	4.05314583714279	5.05334782619827E-05	0.000903285923932941	Scardovia_denticolens	yes
7094.12566311765	4.78444315433818	1.11898248848295	4.27570869390875	1.90530223780536E-05	0.00045409703334361	Lautropia_dentalis	yes
20059.0902827755	6.56433615729243	0.977793200120159	6.7134197256493	1.90115227746434E-11	2.718647756774E-09	Ottowia_massiliensis	yes

Suppl_Table_4_Maaslin2_HUMANn_severity_coeftab

feature	metadata	coef	stderr	pval	qval	group
PWY.5136..fatty.acid..beta..oxidation.II..plant.peroxisome.	Stage_of_severity_of_periodontitis	3.82293675009371	1.33853075349059	0.00428921344207504	0.0302032113212784	y
PWY.6562..norspermidine.biosynthesis	Stage_of_severity_of_periodontitis	3.77788190315895	0.225812753534979	7.90350815251429E-63	1.90813268253559E-61	y
PWY.6902..chitin.degradation.II..Vibrio.	Stage_of_severity_of_periodontitis	2.70594635304483	0.0905593266061121	3.54046246271125E-196	1.70953758913772E-194	y
CENTFERM.PWY..pyruvate.fermentation.to.butanoate	Stage_of_severity_of_periodontitis	2.48510042946112	0.0789551963404834	1.92109872037537E-217	1.08221894581146E-215	y
PWY.6590..superpathway.of.Clostridium.acetobutylicum.acidogenic.fermentation	Stage_of_severity_of_periodontitis	2.45796308953537	0.0686404091409605	7.92204685712286E-281	1.33882591885376E-278	y
PWY.6143..CMP.pseudaminate.biosynthesis	Stage_of_severity_of_periodontitis	2.16304931551787	0.198176040274142	9.79583961121898E-28	1.27345914945847E-26	y
GLUDEG.I.PWY..GABA.shunt	Stage_of_severity_of_periodontitis	2.1143638140729	0.180248411513178	8.91662906455298E-32	1.31035679296474E-30	y
PWY.7820..teichuronic.acid.biosynthesis..B..subtilis.168.	Stage_of_severity_of_periodontitis	1.60691901941462	0.148819645307175	3.52697826785319E-27	4.41525427605326E-26	y
PWY.5209..methyl.coenzyme.M.oxidation.to.CO2	Stage_of_severity_of_periodontitis	1.59986846141795	0.409707479615767	9.42627156531797E-05	0.000708017730906105	y
GALACTARDEG.PWY..D.galactarate.degradation.I	Stage_of_severity_of_periodontitis	1.57519147370551	0.171713099985583	4.58366621345352E-20	4.69478539438573E-19	y
GLUCARGALACTSUPER.PWY..superpathway.of.D.glucarate.and.D.galactarate.degradation	Stage_of_severity_of_periodontitis	1.57519147370551	0.171713099985583	4.58366621345352E-20	4.69478539438573E-19	y
PWY.8190..L.glutamate.degradation.XI..reductive.Stickland.reaction.	Stage_of_severity_of_periodontitis	1.31528982128204	0.0629576999924942	6.37862158674071E-97	1.95997645119851E-95	y
UDPNACETYLGALSYN.PWY..UDP.N.acetyl.D.glucosamine.biosynthesis.II	Stage_of_severity_of_periodontitis	1.28959989029337	0.11129823502581	4.80281323600578E-31	6.76396197404147E-30	y
PWY.7332..superpathway.of.UDP.N.acetylglucosamine.derived.O.antigen.building.blocks.biosynthesis	Stage_of_severity_of_periodontitis	1.18319015150704	0.0847952307755032	2.99542522399866E-44	5.95561015124439E-43	y
GLYCOLYSIS.TCA.GLYOX.BYPASS..superpathway.of.glycolysis..pyruvate.dehydrogenase..TCA..and.glyoxylate.bypa	Stage_of_severity_of_periodontitis	-1.03040403864158	0.149697432647171	5.85044602393812E-12	4.94362689022771E-11	n
PWY.561..superpathway.of.glyoxylate.cycle.and.fatty.acid.degradation	Stage_of_severity_of_periodontitis	-1.09218139833782	0.107517349175986	3.04687642880758E-24	3.55118701012746E-23	n
PWY.5022..4.aminobutanoate.degradation.V	Stage_of_severity_of_periodontitis	-1.14059849777619	0.0422747961157267	2.49802864042852E-160	1.05541710058105E-158	n
P221.PWY..octane.oxidation	Stage_of_severity_of_periodontitis	-1.37364067535133	0.222932539482684	7.19737133532156E-10	5.79217026509211E-09	n
PWY0.166..superpathway.of.pyrimidine.deoxyribonucleotides.de.novo.biosynthesis..E..coli.	Stage_of_severity_of_periodontitis	-1.72010348032162	0.113298804097295	4.65307844971471E-52	1.04849367733571E-50	n
PWY.801..homocysteine.and.cysteine.interconversion	Stage_of_severity_of_periodontitis	-1.85481760519519	0.173987361850914	1.55514117753478E-26	1.87727756430984E-25	n

feature	metadata	coef	stderr	pval	qval	group
PWY.1861..formaldehyde.assimilation.II..assimilatory.RuMP.Cycle.	Treatment_of_periodontitis	2.25712271891729	0.251613602261316	2.94927632614314E-19	3.56019785084422E-18	y
PWY.801..homocysteine.and.cysteine.interconversion	Treatment_of_periodontitis	1.9993988340063	0.173987467523203	1.45350712732085E-30	2.33945432873547E-29	y
P221.PWY..octane.oxidation	Treatment_of_periodontitis	1.62048774862069	0.229471073467554	1.64313446285015E-12	1.58679842412386E-11	y
PWY.5675..nitrate.reduction.V..assimilatory.	Treatment_of_periodontitis	1.45041520038574	0.0241818542178235	0	0	y
PWY.5198..factor.420.biosynthesis.II..mycobacteria.	Treatment_of_periodontitis	1.38629436111989	0.43644290663964	0.00149142150274471	0.0105020930818274	y
TCA.GLYOX.BYPASS..superpathway.of.glyoxylate.bypass.and.TCA	Treatment_of_periodontitis	1.34319419286597	0.0390298619094032	1.52902545747086E-259	1.0336212092503E-257	y
PWY.5209..methyl.coenzyme.M.oxidation.to.CO2	Treatment_of_periodontitis	1.145132304303	0.31895739336745	0.000330378745651672	0.00248151146733923	y
PWY.7209..superpathway.of.pyrimidine.ribonucleosides.degradation	Treatment_of_periodontitis	1.06995703290773	0.0907427501036041	4.33826344916049E-32	7.33166522908122E-31	y
PWY.6590..superpathway.of.Clostridium.acetobutylicum.acidogenic.fermentation	Treatment_of_periodontitis	-1.03397783966822	0.0426930879394563	1.40819955246913E-129	5.28857165260629E-128	n
PWY.8131..5.deoxyadenosine.degradation.II	Treatment_of_periodontitis	-1.03802168913628	0.36097805957634	0.00403284516886212	0.0278184013688856	n
CENTFERM.PWY..pyruvate.fermentation.to.butanoate	Treatment_of_periodontitis	-1.05465447523056	0.0488300066897862	1.85577800026495E-103	5.7022996735414E-102	n
PWY.8190..L.glutamate.degradation.XI..reductive.Stickland.reaction.	Treatment_of_periodontitis	-1.05592516388892	0.06081053777383	1.54083713988196E-67	4.00617656369309E-66	n
PWY.7992..superpathway.of.menaquinol.8.biosynthesis.III	Treatment_of_periodontitis	-1.12018988931266	0.0204389004858232	0	0	n
PWY.5838..superpathway.of.menaquinol.8.biosynthesis.I	Treatment_of_periodontitis	-1.24499292163344	0.0204530279978717	0	0	n
PWY.5861..superpathway.of.demethylmenaquinol.8.biosynthesis.I	Treatment_of_periodontitis	-1.27882111706117	0.0246223410846277	0	0	n
DARABCATK12.PWY..D.arabinose.degradation.I	Treatment_of_periodontitis	-1.28375472180371	0.143076687401447	2.89875050190712E-19	3.56019785084422E-18	n
PWY.5677..succinate.fermentation.to.butanoate	Treatment_of_periodontitis	-1.34937100701529	0.154569222351732	2.54939412453245E-18	2.97136280721368E-17	n
HEXITOLDEGSUPER.PWY..superpathway.of.hexitol.degradation..bacteria.	Treatment_of_periodontitis	-1.35063418072101	0.0872211878738882	4.36963873074186E-54	8.68786994700441E-53	n
GALACTITOLCAT.PWY..galactitol.degradation	Treatment_of_periodontitis	-1.3930648676871	0.160589469518815	4.14655803251732E-18	4.67178871663619E-17	n
PWY.6470..peptidoglycan.biosynthesis.V...beta..lactam.resistance.	Treatment_of_periodontitis	-1.43585504028827	0.0920772584148404	7.99348817963479E-55	1.68862437794785E-53	n
PWY.7434..terminal.O.glycans.residues.modification..via.type.2.precursor.disaccharide.	Treatment_of_periodontitis	-1.51162460241166	0.230163231862441	5.11258710104816E-11	4.67041740582238E-10	n
PWY.6562..norspermidine.biosynthesis	Treatment_of_periodontitis	-1.52809227298015	0.0898595884089444	7.49772435698995E-65	1.81016488047329E-63	n
PWY.6748..nitrate.reduction.VII..denitrification.	Treatment_of_periodontitis	-1.73590910004812	0.0769924493281297	1.45384314392222E-112	4.91398982645711E-111	n
PWY1ZNC.1..assimilatory.sulfate.reduction.IV	Treatment_of_periodontitis	-2.24594776451222	0.599968609606691	0.00018151642894337	0.00142680355774091	n
PWY.6143..CMP.pseudaminate.biosynthesis	Treatment_of_periodontitis	-2.55585838576029	0.249497238738353	1.25874983384867E-24	1.77273934933687E-23	n
KETOGLUCONMET.PWY..ketogluconate.metabolism	Treatment_of_periodontitis	-2.66838494470978	0.0986970729438552	5.56358830774332E-161	2.68641835431034E-159	n
